# Intravenous sildenafil citrate and post-cardiac surgery acute kidney injury: a double-blind, randomised, placebo-controlled trial

**DOI:** 10.1016/j.bja.2020.01.030

**Published:** 2020-04-01

**Authors:** Tracy Kumar, Hardeep Aujla, Marcin Woźniak, Will Dott, Nikol Sullo, Lathishia Joel-David, Paolo Pais, Dawn Smallwood, Douglas Miller, Bryony Eagle-Hemming, Ana Suazo Di Paola, Shaun Barber, Cassandra Brookes, Nigel J. Brunskill, Gavin J. Murphy

**Affiliations:** 1 Department of Cardiovascular Sciences and National Institute for Health Research Leicester Biomedical Research Unit in Cardiovascular Medicine, University of Leicester, Clinical Sciences Wing, Glenfield General Hospital, Leicester, UK; 2 University of Nottingham, Royal Derby Hospital, Derby, UK; 3 School of Allied Health Sciences, De Montfort University, Leicester, UK; 4 Leicester Clinical Trials Unit, University of Leicester, Leicester, UK; 5 Department of Infection, Immunity and Inflammation, University of Leicester, Leicester, UK

**Keywords:** acute kidney injury, cardiac surgery, cardiopulmonary bypass, phosphodiesterase type 5 inhibitors, renal protection, sildenafil citrate

## Abstract

**Background:**

This study assessed whether i.v. sildenafil citrate prevented acute kidney injury in at-risk patients undergoing cardiac surgery with cardiopulmonary bypass.

**Methods:**

In a double-blind RCT, adults at increased risk of acute kidney injury undergoing cardiac surgery in a single UK tertiary centre were randomised to receive sildenafil citrate 12.5 mg kg^−1^ i.v. over 150 min or dextrose 5% at the commencement of surgery. The primary outcome was serum creatinine measured at six post-randomisation time points. The primary analysis used a linear mixed-effects model adjusted for the stratification variables, baseline estimated glomerular filtration rate, and surgical procedure. Secondary outcomes considered clinical events and potential disease mechanisms. Effect estimates were expressed as mean differences (MDs) or odds ratios with 95% confidence intervals.

**Results:**

The analysis population comprised eligible randomised patients that underwent valve surgery or combined coronary artery bypass graft and valve surgery, with cardiopulmonary bypass, between May 2015 and June 2018. There were 60 subjects in the sildenafil group and 69 in the placebo control group. The difference between groups in creatinine concentration was not statistically significant (MD: 0.88 μmol L^−1^ [–5.82, 7.59]). There was a statistically significant increase in multiple organ dysfunction scores in the sildenafil group (MD: 0.54 [0.02, 1.07]; *P*=0.044). Secondary outcomes, and biomarkers of kidney injury, endothelial function, and inflammatory cell activation, were not significantly different between the groups.

**Conclusions:**

These results do not support the use of i.v. sildenafil citrate for kidney protection in adult cardiac surgery.

**Clinical trial registration:**

ISRCTN18386427.

**Editor's key points**

• Acute kidney injury is common after cardiac surgery.
• Intravenous sildenafil has been shown to decrease kidney injury after cardiac surgery in a porcine model.
• This clinical trial in human cardiac surgery patients evaluated the effect of i.v. sildenafil on various surrogate markers of renal injury, inflammation, and organ damage; no meaningful beneficial effect was found.
• Intravenous sildenafil is unlikely to be beneficial for this indication, and larger trials are not warranted based on the findings of this study.

Acute kidney injury (AKI) occurs in up to one-third of all patients after cardiac surgery. It is characterised by an acute decline in kidney function as determined by elevations in serum creatinine, and is associated with significant increases in postoperative complications and an almost four-fold increase in the risk of postoperative death.^1^^,^^2^ Our understanding of the underlying processes is poor and there are no effective preventive measures.^3^ Experimental studies have demonstrated that preservation of endogenous nitric oxide (NO) bioavailability is renoprotective in response to a variety of injurious stimuli.^4^^,^^5^ Endogenous NO activity is increased by the administration of the phosphodiesterase (PDE) type 5 inhibitor sildenafil citrate. This is used clinically in the treatment of erectile dysfunction (Viagra®; Pfizer, New York, NY, USA) and more recently as an i.v. formulation (Revatio®; Pfizer) for pulmonary hypertension and acute right ventricular failure.^6^^,^^7^ We have shown that i.v. sildenafil prevents post-cardiopulmonary bypass (CPB) AKI in a preclinical swine model.^8^ We have also shown that sildenafil administered at a dose of 12.5 mg to adults during cardiac surgery is well tolerated and achieves plasma concentrations known to be clinically effective in some settings.^9^ This Phase IIb efficacy trial tested the hypothesis that the administration of i.v. sildenafil would reduce postoperative AKI in cardiac surgery patients identified before operation as being at increased risk of developing kidney injury.

## Methods

### Trial design/participants

The effect of sildenafil (REVATIO®) on post-cardiac surgery acute kidney injury: a randomised, placebo-controlled clinical trial: the REVAKI-2 trial (registration ISRCTN18386427 on October 1, 2015) was a parallel group RCT conducted at a single tertiary cardiac surgery centre in the UK. Male and female adult patients undergoing coronary artery bypass graft (CABG), open valve, or combined CABG and open-valve surgery who were at increased risk of developing AKI, as determined by a modified AKI risk score^1^ (a predicted risk score of 20% equates to a positive predicted value for developing AKI of >55%) were eligible. Exclusions, listed in the Supplementary material, included patients with pre-existing AKI, sepsis, Stage 5 chronic kidney disease, severe hepatic impairment, allergy to PDE Type 5 inhibitors, or recent treatment with cytochrome P450 3A4 inhibitors or guanylate cyclase stimulators. Participants provided written informed consent before operation. The allocated intervention was administered at skin incision at the start of surgery. Participants were followed-up until discharge and at 6 weeks and 3 months after randomisation. The trial complied with the Declaration of Helsinki. The Yorkshire and The Humber Leeds East Research Ethics Committee approved the study (reference 15/YH/0489) on December 7, 2015. A detailed protocol has been reported elsewhere.^10^ The University of Leicester was the trial sponsor. Changes to the trial after commencement are described in the Supplementary material.

### Randomisation and blinding

Participants were randomly assigned to either sildenafil or placebo in a 1:1 ratio, using an internet-based randomisation system (Sealed Envelope Ltd, a Medicines and Healthcare Products Regulatory Agency recognised facility) with concealed allocation. Randomisation was stratified by (i) type of procedure: CABG, valve, CABG and valve, and other; and (ii) baseline estimated glomerular filtration rate (eGFR): <60, ≥60. Randomisation occurred before operation after written informed consent was given and eligibility confirmed and as close to the scheduled surgery time as possible. Patients, researchers, and clinical staff were blinded to group allocation.

### Interventions

Sildenafil citrate 12.5 mg in dextrose 5% (65 ml) i.v. over 150 min starting at the time of skin incision. In the placebo group, the same volume of a dextrose solution 5% was administered over 150 min. Trial interventions were administered via clear syringes marked only with the participant's trial number and the label *REVAKI-2 trial drug*. Details of perioperative care protocols, monitoring of protocol compliance, blinding of clinical staff, and other steps to mitigate bias are described in the Supplementary material.

### Outcomes

Timings of outcome assessment are listed in Supplementary Table S3.

#### Primary outcome

The primary outcome for the trial was serum creatinine concentration over time in both groups. The time points included were pre-operation (baseline); on arrival to cardiac ICU (CICU) after surgery; and at 6–12, 24, 48, 72, and 96 h post-surgery.

#### Secondary outcomes

Secondary outcomes are listed in Supplementary Table S1. Briefly, these included serial measures of the eGFR and multiple organ dysfunction scores (MODS; range: 0–24, with higher scores indicating more severe organ dysfunction, as defined in a recent trial)^11^ from baseline to 96 h post-surgery, with a final serum creatinine sample for estimation of eGFR at 6 weeks; consensus clinical definitions of acute kidney, lung, liver, gut, brain, and myocardial injury; and sepsis, death, and a composite of these outcomes. Biomarkers of the inflammatory response (serum interleukin [IL]-6, IL-8, and IL-10) and myocardial injury (Serum troponin I), and urine biomarkers of inflammation (neutrophil gelatinase-associated lipocalcin [NGAL]) and injury (tissue inhibitor metalloproteinase-2∗ insulin-like growth factor [IGF]-binding protein-7 [Timp2∗IGFBP7]) were measured in serial serum and urine samples, as described in Supplementary Table S2. Adverse events were reported descriptively. The results of a pre-specified mechanistic analysis of platelet leucocyte and endothelial activation will be reported separately.

### Sample size

On the basis that the observed standard deviation (sd) for serum creatinine values from the MaRACAS study (an observational case control study to identify the role of microvesicle and microvesicle derived micro-RNA in post cardiac surgery acute kidney injury) (NCT02315183) was 37 μmol L^−1^, and the mean observed correlation between baseline and five post-surgery measures of 0.84, we estimated that a sample size of 56 patients per group would have a 90% power to detect a mean difference of 10 μmol L^−1^ for serum creatinine values between treatment and placebo groups with an α value of 0.05, after adjustment for baseline values. We aimed to recruit 126 patients (63 per group), anticipating that 10% of patients would be treated outside of the protocol, withdrawn, or lost to follow-up. This sample size would also allow us to detect an absolute reduction in the frequency of AKI from 65% to 40% with an 80% power and 5% significance (two-tailed).

### Statistical analysis

The primary analysis population included all randomised participants, excluding patients who did not undergo surgery or provide baseline eGFR. Outcomes are reported by intention to treat and were directed by a pre-specified statistical analysis plan (appended to the Supplementary material). Continuous variables are summarised using the mean and sd (or median and inter-quartile range [IQR] if the distribution is skewed), and categorical data are summarised as a number and percentage. The primary analysis of the primary outcome compared all available data with no imputation using a linear mixed-effects model adjusted for the stratification variables, baseline eGFR, and surgical procedure. Treatment effect was estimated with placebo as the reference group, and reported with a 95% confidence interval. A likelihood ratio test was used to determine statistical significance, and two-tailed *P*-values <0.05 were considered statistically significant. The overall treatment effect for the primary outcome was estimated across the post-surgery time points with adjustment for baseline value. A model with time∗treatment interaction effect estimate was also fitted. Significant differences either in the overall effect or for individual time points were considered evidence of a treatment effect. Subgroup analyses considered the interaction between the treatment effect and stratification variables.

Analyses of secondary outcomes were carried out using linear mixed-effects models (for continuous variables that were measured at multiple time points), linear regression models (for continuous variables with a single measure), logistic models (binary variables), or Cox proportional-hazards models (time-to-event variables), as described previously (adjusted for the stratification variables, baseline eGFR, and surgical procedure). All analyses were performed in Stata version 15.0 (StataCorp LP, College Station, TX, USA).

## Results

### Trial cohort and patient flows

**Fig. 1:**
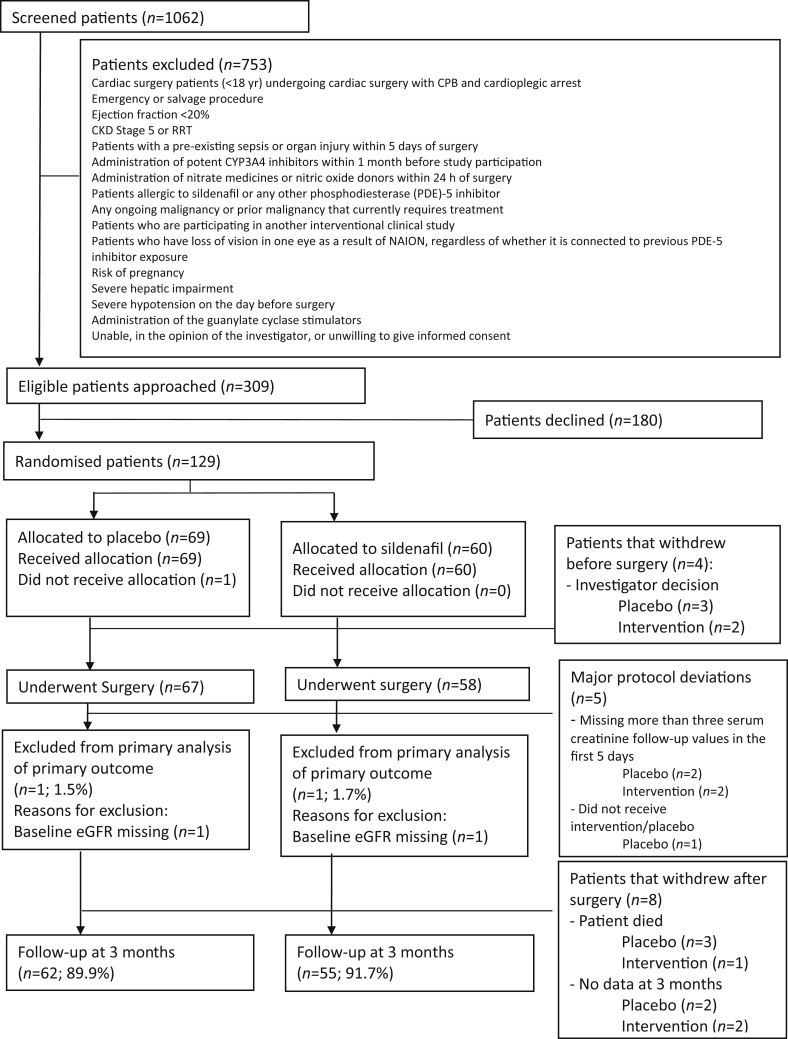
Flow of participants showing eligibility, recruitment, protocol deviations, withdrawals, and loss to follow-up in the REVAKI-2 trial. CKD, chronic kidney disease; CYP3A4, cytochrome P450 3A4; eGFR, estimated glomerular filtration rate; NAION, non-arteritic anterior ischaemic optic neuropathy; RRT, renal replacement therapy.

The flow of patients through the trial is shown in Fig 1. There were 129 patients recruited and randomised between September 2015 and September 2018, which made up the analysis population, 60 of whom were allocated to sildenafil and 69 to placebo. Of the 129 randomised patients, four patients did not undergo surgery and four patients died before hospital discharge. Follow-up was complete for 117 patients (93.6%) at 3 months. Details of patient withdrawals are listed in Supplementary Table S3.

### Participant characteristics

Baseline patient, clinical, and operative characteristics were similar in the two groups (Table 1 and Supplementary Table S4). The median AKI risk score was 29.6 (IQR: 24.5–38) in the sildenafil group and 30.1 (IQR: 25.8–40) in the placebo group. The median age of participants was 72 yr (IQR: 52–88) and 81% were males. Overall, 42 (33.60%) participants were listed for CABG surgery, 39 (31.20%) for valve surgery, and 44 (35.20%) were listed for combined CABG and valve surgery or other. At baseline, the mean eGFR was 70.7 ml min^−1^ m^2^ (1.73)^−2^ in the sildenafil group and 75.6 ml min^−1^ m^2^ (23.6) in the placebo group. By chance, a higher proportion of participants in Sildenafil group 4 (6.90%) underwent redo surgery *vs* Placebo group 2 (2.99%). This was reflected in longer bypass (sildenafil median: 1.76 h [1.36–2.17] *vs* placebo median: 1.58 h [1.17–2.03]) and cross-clamp times (sildenafil median: 1.19 h [0.82–1.57] *vs* placebo median: 1.03 h [0.75–1.27]).

**Table 1:** Participant characteristics and past history. CCS, Canadian Cardiovascular Society; CVA, cerebrovascular accident; CYP3A4, cytochrome P450 3A4; IQR, inter-quartile range; LV, left ventricular; NYHA, New York Heart Association; sd, standard deviation; TIA, transient ischaemic attack.

Characteristic	Placebo (*n*=67)		Sildenafil (*n*=58)		Overall (*n*=125)	
	*n*	%	*n*	%	*n*	%
Demography						
Sex (female)	10	14.93	14	24.14	24	19.2
Age (yr) (mean, range)	72	(52, 88)	72	(54, 88)	72	(52, 88)
BMI (median, IQR)	30.9	(26.6, 36.3)	31.1	(27.7, 35.4)	31	(27.1, 35.7)
AKI risk score (median, IQR)	30.1	(25.8, 40)	29.6	(24.5, 38)	29.95	(25.6, 39.8)
Cardiac disease						
NYHA class						
I	7	10.45	7	12.07	14	11.20
II	50	74.63	37	63.79	87	69.60
III	8	11.94	11	18.97	19	15.20
IV	1	1.49	0	0.00	1	0.80
Missing	1	1.49	3	5.17	4	3.20
CCS class						
Asymptomatic	21	31.34	19	32.76	40	32.00
I	27	40.30	19	32.76	46	36.80
II	15	22.39	15	25.86	30	24.00
III	1	1.49	3	5.17	4	3.20
IV	1	1.49	0	0.00	1	0.80
Missing	2	2.99	2	3.45	4	3.20
LV function						
Good (>49%)	47	70.15	37	63.79	84	67.20
Moderate (30–49%)	16	23.88	19	32.76	35	28.00
Poor (<30%)	3	4.48	2	3.45	5	4.00
Missing	1	1.49	0	0.00	1	0.80
>50% disease in left main stem	4	5.97	5	8.62	9	7.20
≤50% disease in left main stem	63	94.03	51	87.93	114	91.20
Missing	0	0.00	2	3.45	2	1.60
Coronary disease						
None	24	35.82	12	20.69	36	28.80
Number of vessels						
Single	14	20.90	12	20.69	26	20.80
Double	5	7.46	12	20.69	17	13.60
Triple	23	34.33	20	34.48	43	34.40
Missing	1	1.49	2	3.45	2	2.40
Blood and urine results						
Haemoglobin (mean, sd)	128.8	20.1	129.6	17.5	129.2	18.8
Haematocrit (mean, sd)	37.8	5.5	38.3	4.8	38.0	5.2
Platelets (median, IQR)	203	(168, 245)	194	(164.5, 237.5)	196	(166, 238)
Serum creatinine (median, IQR)	89	(73, 103)	92	(79, 110)	90	(75, 106)
Estimated glomerular filtration rate (mean, sd)	75.6	23.6	70.7	20.1	73.3	22.1
Medical history						
Diabetes mellitus						
Yes	24	35.82	26	44.83	50	40.00
No	42	62.69	32	55.17	74	59.20
Missing	1	1.49	0	0.00	1	0.80
*Diet*						
Yes	4	5.97	3	5.17	7	5.60
Not applicable	43	64.18	32	55.17	75	60.00
Missing	20	29.85	23	39.66	43	34.40
*Oral*						
Yes	14	20.90	17	29.31	31	24.80
Not applicable	43	64.18	32	55.17	75	60.00
Missing	10	14.93	9	15.52	19	15.20
*Insulin*						
Yes	7	10.45	7	12.07	14	11.20
Not applicable	43	64.18	32	55.17	75	60.00
Missing	17	25.37	19	32.76	36	28.80
Pacemaker						
Yes	4	5.97	5	8.62	9	7.20
No	62	92.54	53	91.38	115	92.00
Missing	1	1.49	0	0.00	1	0.80
*Temporary*						
Yes	0	0.00	0	0.00	0	0.00
*Permanent*						
Yes	1	1.49	5	8.62	6	4.80
Not applicable	63	94.03	53	91.38	116	92.80
Missing	3	4.48	0	0.00	3	2.40
CVA or TIA						
Yes	2	2.99	1	1.72	3	2.40
No	64	95.52	56	96.55	120	96.00
Missing	1	1.49	1	1.72	2	1.60
Smoking status						
Current	5	7.46	2	3.45	7	5.60
Never	15	22.39	17	29.31	32	25.60
Ex (>1 month)	46	68.66	38	65.52	84	67.20
Missing	1	1.49	1	1.72	2	1.60
Redo cardiac surgery						
Yes	2	2.99	4	6.90	6	4.80
No	65	97.01	54	93.10	119	95.20
Myocardial infarction						
Yes	8	11.94	12	20.69	20	16.00
No	58	86.57	46	79.31	104	83.20
Missing	1	1.49	0	0.00	1	0.80
Medications						
Nitrates until operating theatre						
Yes	2	2.99	3	5.17	5	4.00
No	4	5.97	3	5.17	7	5.60
Not applicable	61	91.04	52	89.66	113	90.40
Clexane® within 12 h before operation						
Yes	0	0.00	0	0.00	0	0.00
No	6	8.96	6	10.34	12	9.60
Not applicable	61	91.04	52	89.66	113	90.40
Anti-platelet agents and dual anti-platelet for 5 days before operation						
Yes	5	7.46	2	3.45	7	5.60
No	1	1.49	4	6.90	5	4.00
Not applicable	61	91.04	52	89.66	113	90.40
CYP3A4 inhibitors within last month						
Yes	0	0.00	0	0.00	0	0.00
No	6	8.96	6	10.34	12	9.60
Not applicable	61	91.04	52	89.66	113	90.40

### Measures of process

Two patients in the placebo group had the infusion stopped prematurely because of hypotension. One participant in the intervention group was found ineligible intraoperatively (ejection fraction <30%) and did not receive the allocated treatment (Supplementary Table S5). Diastolic, systolic, and mean arterial blood pressure; haematocrit; blood loss; transfusion; and change in body weight between baseline and 3 days post-surgery were similar in the sildenafil and placebo groups (Supplementary Fig. S1 and Table S4). The rates of hypotension requiring treatment with vasopressors were similar (30 [52%] sildenafil *vs* 32 [47%] placebo). There were no anaphylactic reactions to the study medication. All participants were alive at the end of the surgery.

### Primary outcome

**Fig. 2:**
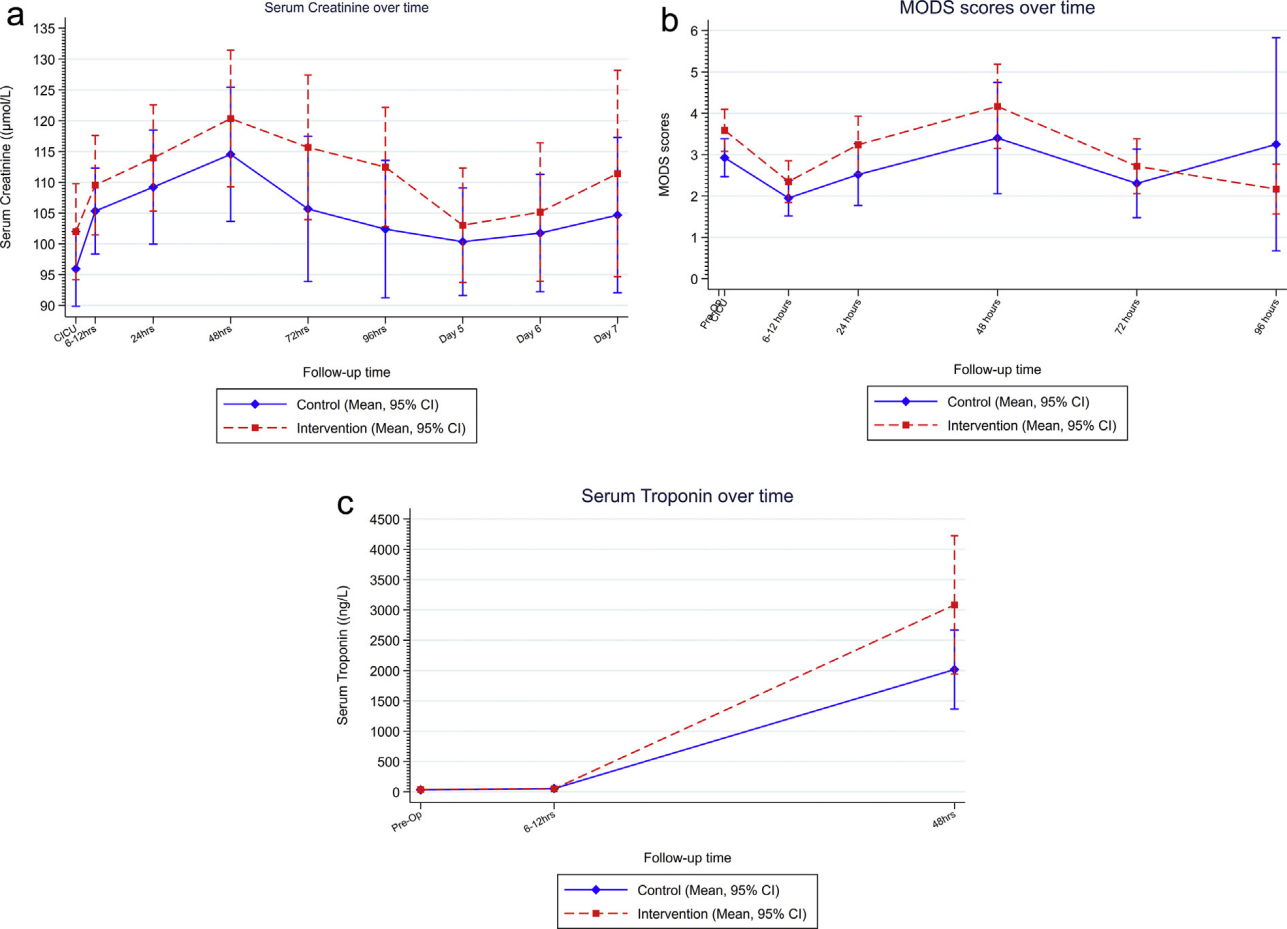
(a) Serum creatinine values. Data expressed as mean (standard deviation [sd]). (b) Multiple organ dysfunction scores. Data expressed as (sd). (c) Serum troponin. Data expressed as mean (sd). CI, confidence interval; CICU, cardiac ICU.

Table 2 and Fig 2a show the results of the analyses of the primary outcome. For the primary intention-to-treat analysis, sildenafil did not reduce serum creatinine up to 96 h after surgery (mean difference: 0.88 μmol L^−1^ [–5.82 to 7.59]; *P*=0.797). No pre-specified secondary, sensitivity, or subgroup analyses indicated a treatment effect of sildenafil. A *post hoc* sensitivity analysis that excluded patients undergoing redo procedures did not demonstrate a treatment effect.

**Table 2:** Primary analysis of primary outcome. All treatment estimates are reported with adjustment for baseline values. Raw data expressed as median (inter-quartile range [IQR]). Number of individuals contributing to each analysis by treatment group and overall: overall: 123; placebo: 66; sildenafil: 57. CICU, cardiac ICU.

Analysis	Randomised to placebo (*n*=67)		Randomised to sildenafil (*n*=58)		Time effect	
	Median (μmol L^−1^)	(IQR) (μmol L^−1^)	Median (μmol L^−1^)	(IQR) (μmol L^−1^)	Adjusted mean difference (95% confidence intervals)	*P*-value
Primary intention to treat						
Baseline	89	(73 103)	92	(79 110)		
CICU	91.5	(80 112)	95.5	(79 118)	Reference group	
6–12 h	101	(84 127)	104.5	(84 129)	8.16 (3.32, 13.00)	0.001
24 h	99	(79 127)	106	(87 138)	13.19 (8.36, 18.01)	<0.001
48 h	100	(82 139)	107.5	(86 153)	18.88 (14.05, 23.7)	<0.001
72 h	96.5	(76 117)	108	(88 137)	12.04 (6.96, 17.13)	<0.001
96 h	97	(78 112)	110	(89 131)	6.95 (1.84, 12.06)	0.008
					**Treatment effect**	
					**Adjusted mean difference (95% confidence interval)**	***P*-value**
Intervention (sildenafil)					0.88 (–5.82, 7.59)	0.797

### Secondary outcomes

Figure 2, Supplementary Table S7, and Figures S2 and S3 show the results of the analyses of secondary outcomes. There was no treatment effect for sildenafil on eGFR up to 6 weeks post-surgery. MODS (scale: 0–24) were higher in the sildenafil group (mean difference: 0.54 [0.02–1.07]; *P*=0.044). The composite outcome of sepsis; low cardiac output; and lung, liver, brain, or gut injury was higher in the sildenafil group, but this was not statistically significant (odds ratio: 3.19 [0.82–12.36]; *P*=0.094). Time to extubation (hazard ratio [HR]: 0.80 [0.55–1.17]), CICU discharge (HR: 1.15 [0.79–1.68]), and discharge from the hospital unit (HR: 0.98 [0.68–1.42]) were similar. The groups were similar with respect to biomarker concentrations for inflammation (IL-6, IL-8, and IL-10) and kidney injury (urine NGAL and Timp2∗IGFBP7) (Supplementary Table S12). Serum troponin was higher in the sildenafil group, but this was not statistically significantly different (mean difference: 21 503 μmol L^−1^ [–3557 to 46 564]; *P*=0.091) (Fig 2c). The time to resolution of arterial hyperlactatemia (>2.0 mM) was longer in the sildenafil arm (median: 3 h [1.4–13.27] *vs* placebo median: 1.67 h [1.23–9.8]). Serious expected adverse events to 3 months were similar in the groups (Supplementary Table S13).

## Discussion

### Main findings

The results of the REVAKI-2 trial do not support the hypothesis that sildenafil citrate reduces the severity of post-cardiac surgery AKI. Unexpectedly, sildenafil increased MODS relative to placebo. This was not reflected by significant differences in clinical outcomes or in serum or urine biomarkers of kidney and myocardial injury.

### Strengths and limitations

The REVAKI-2 trial selected an enriched cohort of patients at increased risk of AKI; 48% of participants developed AKI in the placebo group, although this was less than expected. The trial was double blinded with concealed allocation, detailed documentation of process, objective ascertainment of outcomes, and very low levels of attrition. It evaluated, for the first time, an i.v. sildenafil dose with documented pharmacokinetics that aimed to prevent the early phase reduction in endogenous NO bioactivity through therapeutic plasma concentrations of sildenafil and its active metabolite desmethylsildenafil intraoperatively, and in the immediate postoperative period. The short context-sensitive half-time of these substances was thought to minimise augmentation of late NO-mediated oxidative stress that has been documented in animal models of AKI^4^^,^^12^ and as suggested by elevated NO bioavailability at 48 h post-surgery in the current trial. The trial used detailed analyses of the primary outcome and complementary clinical measures and biomarkers of injury and dysfunction in multiple organ systems. The principal limitation of the trial was the use of serum creatinine as the primary outcome. The limited sensitivity and specificity of this biomarker for AKI are well recognised. This is offset by the clinical applicability of changes in serum creatinine in current consensus definitions of AKI^13^ and the ease, accuracy, and reproducibility of its measurement. Combined with similar values for two putative urine biomarkers of AKI (NGAL and Timp2∗IGFBP7), we conclude that sildenafil is very unlikely to have substantial renoprotective effects in cardiac surgery patients. Another limitation is that baseline eGFR was slightly lower and the proportion of patients undergoing redo surgery was higher in the sildenafil group. However, the pre-specified sensitivity analysis, stratified by eGFR at baseline and a *post hoc* subgroup analysis restricted to those patients who underwent first time cardiac surgery, did not demonstrate any treatment effect, supporting our overall conclusion.

### Clinical relevance

The trial has two novel findings: (i) the intervention was designed based on findings in a porcine model of post-CPB, in which AKI is characterised by alterations in NO activity and in which multiple interventions, including sildenafil, that target these processes have been shown to be renoprotective.^8^^,^^14^ Plasma sildenafil concentrations that were therapeutic in the porcine model and are effective in other clinical settings^9^ had no treatment effect in adult cardiac surgery patients. In a younger cohort (median age: 48 yr) undergoing surgery for rheumatic valve disease, Lei and colleagues^15^ demonstrated that NO added to the CPB circuit or inhalational gases for up to 24 h post-surgery significantly reduced AKI. This effect was attributed to the quenching of plasma cell-free haemoglobin released during CPB by NO, an effect that may not be reproduced by PDE inhibition. Conversely, atrial natriuretic peptide (ANP), which acts in part via PDE inhibition, is renoprotective in adults undergoing cardiac surgery when administered for up to several days post-surgery.^16^^,^^17^ A longer duration of sildenafil treatment may have elicited a different result, or other pleiotropic effects of ANP may underlie these observations. (ii) MODS were higher in the sildenafil group. The effect size was small and less than the minimum clinically important difference for MODS specified in a previous trial.^11^ The risk of over-interpretation of this small and probably clinically non-significant difference notwithstanding it is noteworthy that the direction of the treatment effect was driven predominantly by differences in serum bilirubin values. The treatment effects for serum troponin values were also in the direction of injury, similar to the results of an RCT of pre-surgery oral sildenafil in children undergoing cardiac surgery.^18^ These findings are at odds with preclinical studies indicating a cardioprotective effect of sildenafil in ischaemia–reperfusion injury.^19^ This potential safety signal of sildenafil requires further study.

## Conclusions

We did not demonstrate a renoprotective effect for i.v. sildenafil in adult cardiac surgery patients at increased risk of AKI.

## Authors' contributions

Study conception: GJM, MW

Study design: GJM, NJB, MW

Writing of application for funding: GJM, NJB, MW

Study/research conduct: HA, LJ-D, NS, DS, PP, TK, DM, BEH

Data management: SB, ASDP

Statistical analyses: SB, ASDP

Drafting of report: HA, MW, SB, TK, GJM

All of the study authors, external and internal, had full access to all of the data (including statistical reports and tables) in the study, and can take responsibility for the integrity of the data and the accuracy of the analyses. All authors reviewed the report for important intellectual content and approved the final version.

## Declaration of interest

The authors declare that there are no conflicts of interest.

## Funding

10.13039/501100000274British Heart Foundation (RG/13/6/29947), (CH/12/1/29419) to GJM, MW, TK, and HA; 10.13039/100015250Leicester and Bristol National Institute for Health Research Cardiovascular Biomedical Research Units.
