# Supplementary material for: Intravenous sildenafil citrate and post-cardiac surgery acute kidney injury: a double-blind, randomised, placebo-controlled trial
Source: Br J Anaesth. 2020 Apr 1;124(6):693–701. doi: 10.1016/j.bja.2020.01.030 (PMC7271663; doi:10.1016/j.bja.2020.01.030)
Supplement: Multimedia component 1 [file mmc1.docx]

**The Effect of Intravenous Sildenafil Citrate on Post Cardiac Surgery Acute Kidney Injury: A Double Blinded, Randomised, Placebo-controlled, Clinical Trial**

**Authors:** Tracy Kumar,^1*^ Hardeep Aujla,^1*^ Marcin Wozniak,^1^ Will Dott,^1^ Nikol Sullo,^1,4^ Lathishia Joel-David,^1^ Paolo Pais,^1^ Dawn Smallwood,^1,5^ Ana Suazo Di Paola,^2^ Shaun Barber,^2^ Cassandra Brookes,^2^ Nigel J. Brunskill^3^ and Gavin J. Murphy^1,2^ *****Equal first author

# Expanded Methods

## Participants

Participant may enter study if ALL of the following apply:

1. Adult cardiac surgery patients 18 years of age or above undergoing cardiac surgery with cardiopulmonary bypass and cardioplegic arrest.
2. Identified as representing a high risk group for AKI using a modified AKI risk score; a predicted risk score of 20% equates to a positive predicted value for developing AKI of >55%.
3. Female subjects of childbearing potential are not to be pregnant (to be confirmed by urine human chorionic gonadotropin pregnancy test prior to dosing). Women are considered not to be of childbearing potential if they have been surgically sterilised (e.g. tubal ligation, oophorectomy or hysterectomy) or are postmenopausal (defined as serum follicle-stimulating hormone level of ≥30 IU ml^-1^) in the absence of hormone replacement therapy and complete absence of menses for at least 24 consecutive months.
4. Able, in the opinion of the investigator, and willing to give informed consent.

Participant may not enter study if ANY of the following apply:

1. Cardiac surgery patients (<18 years) undergoing cardiac surgery with cardiopulmonary bypass and cardioplegic arrest.
2. Emergency or salvage procedure
3. Ejection fraction <20%
4. CKD Stage 5, defined as eGFR<15ml min^-1^ or renal replacement therapy. (as per the Modified diet in Renal Disease formula ) or renal replacement therapy
5. Patients with a pre-existing sepsis or organ injury defined as documented sepsis, AKI, acute lung injury, myocardial infarction, low cardiac output, liver injury, stroke or pancreatitis within 5 days of surgery.
6. Administration of potent CYP 3A4 inhibitors within 1 month prior to study participation (e.g. HIV protease inhibitors, imidazole antifungals and erythromycin, please see Appendix 1 for a full list of prohibited medications).
7. Administration of nitrate medicines or NO donors (e.g. glyceryl trinitrate or Nicorandil) within 24 hours of surgery.
8. Patients allergic to sildenafil or any other PDE-5 Inhibitor.
9. Any ongoing malignancy or prior malignancy that currently requires treatment.
10. Patients who are participating in another interventional clinical study.
11. Patients who have loss of vision in one eye due to non-arteritic anterior ischaemic optic neuropathy (NAION), regardless of whether it is connected to previous PDE5 inhibitor exposure.
12. Risk of pregnancy
13. Severe hepatic impairment
14. Severe hypotension (blood pressure < 90/50 mmHg) on the day prior to surgery.
15. Administration of the guanylate cyclase stimulators, such as riociguat (see Appendix 1).
16. Unable, in the opinion of the investigator, or unwilling to give informed consent

## Intervention/ Trial drug

### Trial drug

The active trial drug, Revatio®, is the citrate salt of sildenafil, a selective inhibitor of cyclic guanosine monophosphate (cGMP)-specific phosphodiesterase type-5 (PDE5). Each ml of solution contains 1.124 mg sildenafil citrate, 50.5 mg dextrose and water for injection. Sildenafil is manufactured under Good Manufacturing Practice (EU-GMP) by Pfizer Inc.

### Placebo

The placebo consisted of equivalent volumes of 5% glucose, the solution used for drug storage and dilution, in identical syringes marked only with the patients’ initials and trial number. The placebo was stored at room temperature (15°C to 30°C) and protected from sunlight. The placebo was accessible only to those individuals authorised by the Principal Investigator to dispense and prepare the trial medication.

### Treatment Regimen

Patients were screened by the investigators to assess eligibility for entry into the trial. Eligibility was completed by a medically qualified person delegated to do so on the delegation log. This was fully documented in the medical notes prior to consent. Eligible patients undergoing cardiac surgery with CPB who consented to participate were randomised (via blinded Sealed Envelope system) in a 1:1 manner to:

**Sildenafil** 10mg (12.5ml) bolus IV further diluted with 5% glucose to a total volume of 15ml administered over 20 minutes at a rate of 45ml hr^-1^ followed by 2.5mg as a 2 hour continuous infusion, diluted to a total of 50ml in 5% glucose at a rate of 25ml hr^-1^

or

**Placebo** 15ml (equivalent volume of 5% glucose) bolus IV administered over 20 minutes at a rate of 45ml hr^-1^ followed by 50ml 5% glucose administered as a 2 hour infusion at a rate of 25ml hr^-1^

**Drug and Placebo preparations were as follows:**

|  | Bolus (Syringe 1) | | Continuous Infusion (Syringe 2) | |
| --- | --- | --- | --- | --- |
|  | IV Bolus (dose) | IV Bolus (rate) | IV Continuous (dose) | IV Continuous (rate) |
| Treatment | 12.5ml (i.e. 10mg of sildenafil). Diluted with 5% glucose to a total of 15ml  Marked as syringe 1 | 45ml hr^-1^; i.e. to run over 20 minutes | 2.5mg sildenafil as a 2 hour continuous infusion, diluted to a total of 50ml in 5% glucose Marked as syringe 2 | 25ml hr^-1^, i.e. to run over 2 hours |
| Placebo | 15ml 5% glucose  Marked as syringe 1 | 45ml hr^-1^; i.e. to run over 20 minutes | 50ml 5% glucose Marked as syringe 2 | 25ml hr^-1^, i.e. to run over 2 hours |

One unblinded research nurse prepared the active drug or placebo as per randomisation allocation. Both active drug and placebo were labelled as REVAKI-2 sildenafil/placebo and were identical in appearance. This was handed to the blinded clinical team. Patients, clinicians and all other researchers were blinded.

### Deviations from Protocol

In the event of any deviation from the trial protocol, defined as the non- administration of the allocated drug or its administration not in accordance with the trial protocol the deviation was documented and the patient continued to be treated according to the randomised allocation.

### Concomitant Treatment

Patients could receive medications and/or other therapies to treat adverse events as deemed necessary by the investigator or the patient’s physician. Concomitant medications and/or therapy that become necessary during the trial and any changes in concomitant medication and/or therapy was recorded on the CRFs as follows:

- Anti platelet agents, & dual anti platlet therapy for 5 days pre surgery

- Morning of surgery to hospital discharge: All antibiotic regimens

Details of concomitant medications and therapy included generic drug name, dose, route, duration and indication.

### Preoperative Care

Eligible patients received standard care preoperatively as per local practice.

### Anaesthesia and Perioperative care

Local protocols for anaesthesia and perioperative care were used. Anaesthetic maintenance used either volatile or intravenous anaesthetic agents or both in combination.

### Cardiopulmonary bypass

CPB was managed according to local practice. All patients underwent non-pulsatile or pulsatile CPB, (28-35°C). The circuit used a standard or closed venous reservoir, a roller or centrifugal pump, a hollow fibre oxygenator and a heparin or non-heparin bonded circuit. Target flows were 2.4-2.7 L min^-1^ m^2^, with MABP maintained between 60-80mmHg. Circuit prime was not standardised. Intermittent or continuous, antegrade and/or retrograde blood cardioplegic arrest was performed. Haematocrit was maintained >23. Target activated clotting time of >400 seconds was achieved with heparin (300u kg^-1^ as a loading dose) for bypass. Heparin reversal was achieved with the administration of protamine sulphate in a 1:1 ratio as per standard practice.

### Haemodynamic support

The use of inotropes or vasopressors was at the discretion of the attending physician. Blood products were transfused using existing unit protocols. Fluid replacement therapy was as directed according to local practice.

## Assessment of Secondary Outcomes

Urine and serum samples were collected at baseline and postoperatively for the measurement of markers of inflammation, myocardial, and kidney injury. Clinical management of trial patients, anaesthetic technique, and cardiopulmonary bypass were standardised where possible. Briefly, propofol infusion at 3 mg kg^-1^ per hour was combined with remifentanil infusion at 0.5 to 1 μg kg^-1^ per minute. Neuromuscular blockade was achieved by 0.1 to 0.15 mg kg^-1^ pancuronium bromide or vecuronium, and the lungs were ventilated to normocapnia with air and oxygen (45% to 50%). Volatile inhalational drugs were used in addition to propofol at the physicians’ discretion. Mean arterial pressure of 60 mmHg or above was maintained with increments of metaraminol 0.5 to 1.0 mg or volume. Heparin was given at a dose of 300 IU kg^-1^ to achieve a target activated clotting time of 480 seconds or above before commencement of cardiopulmonary bypass. Additional 3000 IU of heparin was administered if required. Cardiopulmonary bypass was established by using conventional atrial and arterial cannulae. The CPB circuit and prime was not standardised however non-pulsatile flow was used, and the target flow rate throughout bypass was 2.4 L m^-2^ per minute. Systemic temperature was actively cooled down to 32°C. Myocardial protection was achieved by using antegrade or retrograde cold (6 to 8°C) blood cardioplegia, with added K+ and Mg2+ to give a final concentration of 20 mmol L^-1^ K+ and 5 mmol L^-1^ Mg2+. Post bypass heparin was reversed in a 1:1 ratio using protamine.

## Data collection

In-hospital data collection included the following elements:

- Screening data for all non-emergency patients having cardiac surgery:
  - - Distinguishing patients but without recording identifiable data electronically
    - Whether or not a Patient Information Leaflet (PIL) was sent
    - Whether or not a patient was approached for the trial
    - Planned operation
    - Assessment of eligibility; if ineligible, reasons for ineligibility
    - Whether or not a patient was asked to give written informed consent for the trial
- For all randomised participants:
  - Pre-operative characteristics
  - Operative details
  - Details of random allocation, including whether this was adhered to
  - Observations required for the primary and secondary outcomes, including dates and times of relevant events
  - Any AEs

Research staff collected data on the trial screening log and pre-printed case report forms (CRFs). These data were transferred promptly to a secure computerised database maintained on a University of Leicester server, allowing data to be checked centrally. Queries about specific data items were listed on the database and were immediately apparent to centre staff when they logged on.

Data collection after hospital discharge consisted of the following elements:

- Six weeks after the operation: Serum creatinine for estimation of eGFR, adverse events recorded.
- Three months after the operation:
  - AEs occurring after discharge, with further details of any event suspected to contribute to the primary outcome or meet the definition of an SAE sought from either the admitting hospital or the participant’s general practitioner (GP).

Serum, urine and plasma samples were stored for biomarker analyses performed in batches at the Department of Cardiovascular Sciences at the University of Leicester. These data, along with laboratory data from samples obtained as part of routine clinical care were linked via data download to the Leicester CTEU for analyses.

The schedule for data collection is summarised in **Table S3**.

## Laboratory analyses

Serum Troponin, Neutrophil Gelatinase Associated Lipocalcin (NGAL), TIMP2*IGFBP, IL6, 8, 10, and NO bioavailability, were measured in all available samples. After collection serum samples were left to clot for at least 30 minutes and then centrifuged at 3500 rpm for 5 minutes. Remaining serum was aliquoted into cryovials, and frozen at -80⁰C. Urine samples were centrifuged at 1500g for 15 minutes, aliquoted into cryovials, and frozen at -80⁰C until analysis. Serum Troponin was measured in the Department of Cardiovascular Sciences at the University of Leicester using established SOPs, using the Enzo® Troponin I (human) ELISA kit (Enzo Life Sciences, Ann Arbor, MI, USA). The lower limit for detection of this assay is 0.38ng ml^-1^. Serum creatinine, was measured on the Advia 2400 Chemistry System (Siemens, Frimley, UK). IL-6 (EASIA kit Invitrogen), IL-8 ELISA, (Bender Medsystem GmbH) NGAL, TIMP2, IGFBP, and IL-10 (all EIA kit, EKF diagnostic) were measured on the DS2® 2-Plate ELISA Processing System (Dynex Technologies, Chantilly, VA).

# Changes to the protocol after commencement of the study

**Inclusion Criteria**

Identified as representing a high risk group for AKI using a modified AKI risk score equal or greater than “22%” changed to “20%”. (Protocol V1.0, dated 20/07/2016 changed to Protocol V2.0, dated 05/06/2017).

**Primary Outcome**

No changes made.

**Secondary Outcomes**

Serum amylase concentration measured for secondary outcomes and expected adverse events “>1000ng ml^-1^” changed to “>1000IU L^-1^”. (Protocol V1.0, dated 20/07/2016 changed to Protocol V2.0, dated 05/06/2017).

Myocardial Injury measured from serum troponin I at “baseline and at 24 hours” changed to “baseline and at 6-12 and 48 hours post-operation”. Approximate blood collection volumes amended accordingly. (Protocol V1.0, dated 20/07/2016 changed to Protocol V2.0, dated 05/06/2017).

We removed tracheal aspirates as a means of measuring platelet, leucocyte and endothelial cell activation. These were still measured in blood samples. (Protocol V2.2, dated 16/04/2018 changed to Protocol V2.3, dated 11/06/2018).

All these changes received REC approval.

# Supplementary Tables

**Table S1. Secondary Outcomes**

| **Outcome** | **Definition/ Method of Verification** |
| --- | --- |
| AKI | Defined according to the KDIGO criteria defined as a rise in serum creatinine of >26µmol L^-1^ within 48 hours or a doubling of the serum creatinine within 7 days of surgery.^1^ |
| Biomarkers of AKI | These included urine NGAL at baseline and at 24 hours. We also calculated absolute change from baseline for serum creatinine. eGFR was estimated in all patients at 6 weeks post-surgery using the Modification of Diet in Renal Disease equation).^2^ |
| Inflammatory Organ Injury, Sepsis or Death | - Sepsis was defined as antibiotic treatment for suspected infection, ***and*** *th*e presence of SIRS within 24 hours prior to start of antibiotic treatment where SIRS is defined as ≥ 2 of the following conditions: temperature > 38 ^o^C or < 36 ^o^C; heart rate > 90 beats min^-1^; respiratory rate > 20 breaths min^-1^ or PaCO2 < 32 mmHg; white blood cell count > 12,000 / mm^3^ or < 4,000 / mm^3^, **or** antibiotic treatment for wound infection. - Acute lung injury, defined as PaO2/FiO2 ratio <300mmHg and CPAP/PEEP of 5 cmH2O (490 kPa).^3^ - Low cardiac output, defined as new intra-or postoperative intra-aortic balloon pump insertion or a cardiac index of <2.2 L min^−1^ m^−2^ refractory to appropriate intravascular volume expansion after correction or attempted correction of any dysrhythmias, or the administration of the inotropes enoximone, milrinone or levosimendan. - Stroke; diagnosed by brain imaging (CT or MRI), in association with new onset focal or generalized neurological deficit (defined as deficit in motor, sensory or co-ordination functions) - Acute liver injury was defined as an acute derangement of liver enzymes three times the upper limit of normal, or an acute derangement of liver enzymes associated with progressive hypoglycaemia and lactate acidosis, or a serum amylase concentration >1000IU L^-1^ from daily blood samples. - Acute intestinal injury was defined a radiological, operative or post-mortem evidence of gut ischaemia. |
| Injury Score | - The Multiple Organ Dysfunction Score ^4^ was calculated at baseline, admission to ICU, 24, 48, 72 and 96 hours post-surgery. |
| Bleeding and Transfusion | - Blood loss at 6 hours postoperatively. - The total number of units of RBC and other blood components transfused during the operative period and post-operative hospital stay was recorded |
| Drug Reactions | Vital sign measurements during and after drug administration to document;   - Hypotensive reactions and interventions - Allergic Reactions. |
| Other adverse events | Other expected and unexpected adverse events not listed above. |
| Hospital stay and cumulative resource use | Time until extubation, discharge from HDU and discharge from hospital were measured from the start of surgery, deaths was censored at time of death. |
| Myocardial Injury | Serum Troponin I at baseline and at 6-12 & 48 hours. |
| Inflammation | Platelet and leucocyte activation was assessed in blood samples collected at baseline an then at 6 and 48 hours post-surgery using flow cytometry. |
| Endothelial injury | Markers of endothelial activation was measured in blood samples taken at baseline and then at 6-12hrs and 48 hours post-surgery using flow cytometry. Regional endothelial dysfunction was measured as the reactive hyperemia peripheral arterial tonometry (RH-PAT) index using the Endo-PAT 2000 (Itamar Medical Ltd., Caesarea, Israel) ^5^. Global endothelial dysfunction was also measured indirectly as the measured time to resolution of oxygen debt defined as the period of time from the end of surgery until the measured serum arterial lactate level falls below 2.5 mmol L^-1^. |

**Table S2: Schedule of data collection**

|  | Pre-Op’n | Op’n Day  +/-12 hours | Day 1  +/-12 hours | Day 2  +/-12hours | Day 3  +/-12hours | Day 4  +/-12hours | Day 5  +/-12hrs | Days 6&7  +/-12hours | 6 wks  +/-3 weeks | 3 months  Postal/medical notes/GP records* |
| --- | --- | --- | --- | --- | --- | --- | --- | --- | --- | --- |
| Eligibility | ✓ |  |  |  |  |  |  |  |  |  |
| Written consent | ✓ |  |  |  |  |  |  |  |  |  |
| Concomitant medication  concomitant medication only in relation to infection apart from pre op | ✓ | ✓ | ✓ | ✓ | ✓ | ✓ | ✓ | ✓ | ✓ | ✓ |
| Pregnancy testing | ✓ |  |  |  |  |  |  |  |  |  |
| Randomisation | ✓ |  |  |  |  |  |  |  |  |  |
| Operative details |  | ✓ † |  |  |  |  |  |  |  |  |
| MODS Scores | ✓ | (6-12hrs) | ✓  (24 hrs) | ✓  (48 hrs) | ✓  (72 hrs) | ✓  (96 hrs) |  |  |  |  |
| Clinical outcomes |  |  | ✓ | ✓ | ✓ | ✓ | ✓ |  | ✓§ | ✓ Ʊ |
| Serious adverse event monitoring/Adverse Events |  | ✓ | ✓ | ✓ | ✓ | ✓ | ✓ | ✓ | ✓§ | ✓ |
| Bloods: serum biochemistry (creatinine, amylase, liver function tests) and full blood counts | ✓ | ✓  (CICU †& 6 -12 hrs) | ✓ † | ✓ † | ✓ † | ✓† | ✓ Λ † | ✓ Λ † | ✓ |  |
| Organ Injury Mark*e*rs; eg Urine NGAL, Serum troponin**.** | Urine ✓ Troponin I | ✓ Troponin I (6-12) | Urine ✓ | ✓ Troponin I (48) |  |  |  |  |  |  |
| RH-PAT testing | ✓ |  | ✓ |  |  |  |  |  |  |  |
| Bloods: Citrated whole blood for flow cytometry | ✓ | ✓  (6 -12 hrs) |  | ✓ |  |  |  |  |  |  |
| Questionnaire |  |  |  |  |  |  |  |  | ✓ | ✓ |

***Notes:***

a. Renal function assessed by serum creatinine and/or urinary output, using KDIGO criteria for acute kidney injury

e. Biochemical markers of heart and kidney injury and markers of systemic inflammation were measured from venous blood taken pre-operatively, on return to ITU and at 6, 24, 48, 72, and 96 hours post-operatively.

f. Biochemical markers of renal injury were measured from urine samples taken pre-operatively and on days 1 and 2 post-operatively, as described in the text.

h. CPB duration, nadir temperature, serial haematocrit, pump flows, lactate, and mixed venous oxygen saturation were recorded.

i. Units and timing of RBC, and units of platelet and fresh frozen plasma transfusions were recorded.

**Table S3. Withdrawals**

|  |  | **Randomised to**  **Placebo (N=69)** | | | **Randomised to Sildenafil (N=60)** | | | **Overall**  **(N=129)** | |
| --- | --- | --- | --- | --- | --- | --- | --- | --- | --- |
|  |  | **N** | **%** | **N** | | **%** | **n** | | **%** |
| **Any withdrawal*** |  | 7 | 10.14 | 5 | | 8.33 | 12 | | 9.30 |
| **Of those who withdrew:** | |  |  |  | |  |  | |  |
| **Time of withdrawal** | After randomisation but before surgery | 2 | 2.90 | 2 | | 3.33 | 4 | | 3.10 |
|  | After surgery | 5 | 7.25 | 3 | | 5.00 | 8 | | 6.20 |
| **Reason for withdrawal** | Death | 3 | 4.35 | 1 | | 1.67 | 4 | | 3.10 |
|  | Investigator’s decision | 2 | 2.90 | 1 | | 1.67 | 3 | | 2.33 |
|  | Missing (did not undergo surgery) | 2 | 2.90 | 3 | | 5.00 | 5 | | 3.88 |

**Table S4: Operative Characteristics and Measures of Process**

| **Characteristic** | |  | **Randomised to Placebo (N=67)** | | **Randomised to Sildenafil (N=58)** | |
| --- | --- | --- | --- | --- | --- | --- |
|  | | | **n** | **%** | **n** | **%** |
| Type of surgery | Isolated CABG | | 22 | 32.84 | 20 | 34.48 |
|  | Other than Isolated CABG | | 45 | 67.16 | 38 | 65.52 |
| Bypass duration (mins) (median, IQR) | |  | 95 | (70,122) | 105.5 | (81.5,130) |
| Cross clamp duration (mins) (median, IQR) | | | 62 | (45,76) | 71.5 | (49,94) |
| **VALVE SURGERY** | |  | 22 | 32.84 | 17 | 29.31 |
| **Other Surgery** | |  |  |  |  |  |
| Aortic Procedure | |  | 4 | 5.97 | 3 | 5.17 |
| Correction of congenital abnormality | |  | 0 | 0.00 | 0 | 0.00 |
| Other | |  | 0 | 0.00 | 2 | 3.45 |
| **Transfusion** | |  |  |  |  |  |
| Total RBCs units transfused (median, IQR) | |  | 2 | (2,4) | 3 | (2,6) |
| Any RBC units transfused | |  | 37 | 55.22 | 35 | 60.34 |
| Total FFP units transfused (median, IQR) | |  | 2 | (1.5,2) | 2 | (1.5,4) |
| Any FFP units transfused | |  | 19 | 28.36 | 20 | 34.48 |
| Total Platelets transfused (median, IQR) | |  | 1 | (1,1) | 1 | (1,2) |
| Any Platelets transfused | |  | 18 | 26.87 | 19 | 32.76 |
| Total Cryoprecipitate (median, IQR) | |  | 1 | (1,2) | 2 | (1,2) |
| Any Cryoprecipitate transfused | |  | 17 | 25.37 | 18 | 31.03 |
| **Mean Arterial Blood Pressure (median, IQR)** | |  |  |  |  |  |
| Start of CPB | |  | 60 | (54,66) | 55 | (50,60) |
| 20 minutes after the start of CPB | |  | 60 | (55,66) | 55 | (52,63) |
| 40 minutes after the start of CPB | |  | 60 | (54,67) | 58 | (52,70) |
| Pre-warm | |  | 65.5 | (55.5,71) | 60.5 | (55,66) |
| Pre-wean | |  | 60 | (56,67) | 57.5 | (52,65) |
| **Hypotension** | |  | 32 | 47.76 | 30 | 51.72 |
| **Hypotension with rash** | |  | 0 | 0.00 | 0 | 0.00 |
| **Body mass at baseline (median, IQR)** | |  | 30.9 | (26.6,36.3) | 31.1 | (27.7,35.4) |
| **Body mass at 72 hours (median, IQR)** | |  | 32.5 | (28.6,36.8) | 31.5 | (28.4,36.5) |

**Table S5: Protocol deviations**

|  | **Randomised to**  **Placebo (N=69)** | | **Randomised to**  **Sildenafil (N=60)** | | **Overall**  **(N=129)** | |
| --- | --- | --- | --- | --- | --- | --- |
|  | **N** | **%** | **N** | **%** | **N** | **%** |
| Underwent randomisation but did not undergo surgery | 2 | 2.90 | 2 | 3.33 | 4 | 3.10 |
| 3 month follow-up not completed | 7 | 10.14 | 5 | 8. 33 | 12 | 9.30 |
| Ineligible but treated | 0 | 0.00 | 0 | 0.00 | 0 | 0.00 |
| Did not receive an intervention for participants that underwent surgery | 1 | 1.49 | 0 | 0.00 | 1 | 0.80 |
| >3 missing values for the primary outcome in the first 5 days | 2 | 2.90 | 2 | 3.33 | 4 | 3.10 |
| Total Major Protocol Violations | 3 | 4.35 | 2 | 3.33 | 5 | 3.88 |

Major protocol deviations were defined in the Statistical Analysis Plan as not receiving the allocated intervention or >3 missing values for the primary outcome.

**Table S6: Secondary and Sub-group analyses of Primary Outcome**

|  | **Adjusted Mean Difference**  **(95% Confidence Intervals)** | **P Value** |
| --- | --- | --- |
| **Secondary Analyses** | **Treatment effect** |  |
| Model including Time by Treatment Interaction | 0.93 (-6.65, 8.50) | 0.810 |
|  | *Time (hours)x Intervention Interaction:*  0.00 (-0.10, 0.10) | 0.971 |
| Linear Regression of highest Serum Creatinine value | 1.00 (0.92, 1.09) | 0.966 |
| Model Including only creatinine values collected within specified time windows | 1.03 (-3.75,5.82) | 0.672 |
| Model of Area Under Serum Creatinine Curve | 421.4 (-592.06,1434.86) | 0.410 |
| **Sub-Group Analyses** |  |  |
| Isolated CABG | 8.09 (-5.21,21.39) | 0.233 |
| Other Surgery types (not Isolated CABG) | -3.81 (-11.93, 4.30) | 0.357 |
| Renal Impairment (baseline eGFR<60ml min^-1^) | 3.52 (-14.26, 21.31) | 0.698 |
| No Renal Impairment (baseline eGFR≥60ml min^-1^) | -0.30 (-6.95, 6.35) | 0.929 |
| No Pre-operative diagnosis of diabetes | -3.56 (-11.26, 4.14) | 0.365 |
| Pre-operative diagnosis of diabetes | 3.73 (-8.12,15.58) | 0.537 |
| No previous Cardiac Surgery | -0.58 (-7.73,6.57) | 0.874 |
| Previous Cardiac Surgery | -18.89 (-46.28,8.49) | 0.176 |

*Number of individuals contributing to each analysis by treatment group and overall:*

**Model including time by treatment interaction:** Overall: 122, Sildenafil: 57, Placebo: 65

**Linear Regression of highest Serum Creatinine value:** Overall: 123, Sildenafil: 57, Placebo: 66

**Model including only creatinine values collected within specified time windows:** Overall: 122, Sildenafil: 57, Placebo: 65

**Model of Area Under Serum Creatinine Curve:** Overall: 86, Sildenafil: 43, Placebo: 43

**Sub-group of Isolated CABG:** Overall: 41, Sildenafil: 20, Placebo: 21

**Sub-group of Other Surgery types (not Isolated CABG):** Overall: 82, Sildenafil: 37, Placebo: 45

**Sub-group of Renal Impairment (baseline eGFR<60ml min^-1^):** Overall: 31, Sildenafil: 16, Placebo: 15

**Sub-group of No Renal Impairment (baseline eGFR≥60ml min^-1^):** Overall: 92, Sildenafil: 41, Placebo: 51

**Sub-group of No Pre-operative diagnosis of diabetes:** Overall: 73, Sildenafil: 31, Placebo: 42

**Sub-group of Pre-operative diagnosis of diabetes**: Overall: 49, Sildenafil: 26, Placebo: 23

**Sub-group of No previous Cardiac Surgery:** Overall: 112, Sildenafil: 49, Placebo: 63

**Sub-group of Previous Cardiac Surgery:** Overall: 10, Sildenafil: 8, Placebo: 2

**Table S7: Secondary outcomes**

|  | **Randomised to Placebo (N=67)** | | **Randomised to Sildenafil (N=58)** | | **Overall**  **(N=125)** | | **Effect (95% CI)** | **p-value** |
| --- | --- | --- | --- | --- | --- | --- | --- | --- |
|  |  |  |  |  |  |  |  |  |
| **eGFR (mean, SD)** |  |  |  |  |  |  |  |  |
| Baseline | 75.6 | 23.6 | 70.7 | 20.1 | 73.3 | 22.1 | - | - |
| CICU | 72.9 | 20.6 | 68.0 | 22.4 | 70.6 | 21.5 | Reference | - |
| 6-12 hours | 67.4 | 21.9 | 62.4 | 20.2 | 65.1 | 21.2 | -5.35 (-8.37,-2.33) | 0.001 |
| 24 hours | 67.6 | 24.7 | 60.4 | 21.8 | 64.2 | 23.6 | -6.87 (-9.88,-3.86) | 0.000 |
| 48 hours | 65.9 | 25.5 | 58.4 | 22.2 | 62.4 | 24.2 | -8.41 (-11.43,-5.39) | 0.000 |
| 72 hours | 71.2 | 27.3 | 62.1 | 26.6 | 66.8 | 27.2 | -3.81 (-6.96,-0.65) | 0.018 |
| 96 hours | 72.4 | 25.4 | 61.1 | 23.7 | 67.0 | 25.1 | -2.49 (-5.66,0.69) | 0.124 |
| Day 5 | 71.8 | 24.2 | 63.9 | 23.5 | 68.4 | 24.1 | -1.80 (-5.11,1.52) | 0.288 |
| Day 6/Discharge | 69.3 | 20.0 | 66.6 | 22.3 | 68.1 | 20.9 | -1.54 (-5.10,2.02) | 0.397 |
| Day 7/Discharge | 68.3 | 23.9 | 65.6 | 24.5 | 67.2 | 23.9 | -0.97 (-4.81,2.88) | 0.622 |
| 6 weeks | 66.3 | 19.3 | 61.4 | 17.3 | 63.9 | 18.4 | -6.07 (-9.27,-2.86) | 0.000 |
| Intervention (SILDENAFIL) |  |  |  |  |  |  | -4.33 (-10.04,1.37) | 0.137 |
| **Multiple Organ Dysfunction Score (median, IQR)** |  |  |  |  |  |  |  |  |
| Baseline | - | - | - | - | - | - | - | - |
| CICU | 3 | (2,4) | 4 | (2,5) | 3 | (2,4) | Reference | - |
| 6-12 hours | 1 | (1,3) | 2 | (1,3) | 2 | (1,3) | -1.07 (-1.39,-0.74) | 0.000 |
| 24 hours | 2 | (1,4) | 3 | (2,4) | 3 | (1.5,4) | -0.72 (-1.14,-0.30) | 0.001 |
| 48 hours | 3 | (2,4) | 4 | (3.5,5) | 4 | (2,4) | -0.31 (-0.90,0.28) | 0.303 |
| 72 hours | 2 | (1,4) | 2 | (2,4) | 2 | (1,4) | -0.91 (-1.35,-0.48) | 0.000 |
| 96 hours | 3 | (1,5.5) | 2 | (2,3) | 2 | (1,3) | -1.71 (-2.54,-0.88) | 0.000 |
| Intervention (SILDENAFIL) |  |  |  |  |  |  | 0.54 (0.02,1.07) | 0.044 |
| **NO Bioavailability (median, IQR)** |  |  |  |  |  |  |  |  |
| Baseline | 73.6 | (58.8,96.2) | 74.5 | (63.1,88.7) | 74.2 | (61.4,94.2) | Reference | - |
| 6-12 hours | 74.6 | (65.8,86.8) | 70.5 | (61.2,81.1) | 71.2 | (64,85.9) | -2.44 (-9.20,4.32) | 0.479 |
| 48 hours | 95.6 | (79.3,119.3) | 98.9 | (82,127.4) | 96.5 | (79.9,121.3) | 22.39 (15.41,29.38) | 0.000 |
| Intervention (SILDENAFIL) |  |  |  |  |  |  | -0.39 (-8.10,7.32) | 0.920 |
| **LENGTH OF STAY (median, IQR)** | |  |  |  |  |  |  |  |
| Hospital Unit length of stay (Hours) | 194.5 | (145.5,266.8) | 195.1 | (150.1,266.3) | 194.9 | (148.3,266.3) | 1.18 (0.81, 1.73) | 0.394 |
| Hospital Unit length of stay (Days) | 8.1 | (6.1,11.1) | 8.1 | (6.3,11.1) | 8.1 | (6.2,11.1) | 1.18 (0.81, 1.73) | 0.394 |
| **CLINICAL OUTCOMES** |  |  |  |  |  |  |  |  |
| Composite of Organ Injury, Sepsis or Death | 53 | 79.10 | 52 | 89.66 | 105 | 84.00 | 3.19 (0.82 , 12.36) | 0.094 |
| Sepsis | 3 | 4.48 | 2 | 3.45 | 5 | 4.00 | 0.76 (0.12 , 4.95) | 0.773 |
| Brain Injury | 1 | 1.49 | 2 | 3.45 | 3 | 2.40 | 1.92 (0.14 , 26.59) | 0.627 |
| Low cardiac Output | 2 | 2.99 | 2 | 3.45 | 4 | 3.20 | 1.04 (0.13 , 8.50) | 0.970 |
| Acute kidney injury (binary) | 33 | 49.25 | 25 | 43.10 | 58 | 46.40 | 0.67 (0.32 , 1.42) | 0.297 |
| Acute Kidney injury (ordinal) |  |  |  |  |  |  | 0.62 (0.30 , 1.28) | 0.197 |
| Acute Kidney injury (stage 1) | 25 | 37.31 | 21 | 36.21 | 46 | 36.80 |  |  |
| Acute Kidney injury (stage 2) | 4 | 5.97 | 3 | 5.17 | 7 | 5.60 |  |  |
| Acute Kidney injury (stage 3) | 4 | 5.97 | 1 | 1.72 | 5 | 4.00 |  |  |
| Acute Lung Injury | 47 | 70.15 | 47 | 81.03 | 94 | 75.20 | 1.62 (0.68 , 3.82) | 0.275 |
| Acute Liver Injury | 7 | 10.45 | 2 | 3.45 | 9 | 7.20 | 0.29 (0.06 , 1.49) | 0.138 |
| Acute Intestinal Injury | 5 | 7.46 | 5 | 8.62 | 10 | 8.00 | 1.17 (0.31 , 4.45) | 0.817 |
| Death within 90 days of surgery | 3 | 4.48 | 1 | 1.72 | 4 | 3.20 | 0.29 (0.02 , 3.55) | 0.332 |
| **BMI AT 72 HOURS (median, IQR)** |  |  |  |  |  |  |  |  |
| Baseline | 32.5 | (28.6 , 36.8) | 31.5 | (28.4 , 36.5) | 31 | (27.1 , 35.7) | 0.89 (0.85 , 0.94) | 0.000 |
| Intervention (SILDENAFIL) |  |  |  |  |  |  | 0.41 (-0.22 , 1.04) | 0.198 |

*Number of individuals contributing to each model by group and overall:*

**eGFR:** Overall: 123, Sildenafil: 57, Placebo: 66.

**MODS:** Overall: 121, Sildenafil: 55, Placebo: 66.

**NO Bioavailability:** Overall: 123, Sildenafil: 57, Placebo: 66.

**Hospital Unit length of Stay:** Overall: 119, Sildenafil: 53, Placebo: 66.

**Clinical Outcomes:**

- **Sepsis:** Overall: 113, Sildenafil: 53, Placebo: 60.
- **Brain Injury:** Overall: 117, Sildenafil: 55, Placebo: 62.
- **Low Cardiac Output:** Overall: 112, Sildenafil: 51, Placebo: 61.
- **Acute Kidney Injury:** Overall: 119, Sildenafil: 55, Placebo: 64.
- **Acute Lung Injury:** Overall: 121, Sildenafil: 57, Placebo: 64.
- **Composite of Organ Injury, Sepsis or Death:**

Overall: 116, Sildenafil: 54, Placebo: 62.

- **Death within 90 days of surgery:**

Overall: 123, Sildenafil: 57, Placebo: 66.

- **BMI at 72 hours:** Overall: 110, Sildenafil: 52, Placebo: 58.

**Table S12: Serum and urine biomarkers of inflammation and organ injury**

|  | **Randomised to Placebo (N=67)** | | **Randomised to Sildenafil**  **(N=58)** | | **Overall**  **(N=125)** | | **Effect**  **(95% CI)** | **p-value** |
| --- | --- | --- | --- | --- | --- | --- | --- | --- |
|  | **Median** | **IQR** | **Median** | **IQR** | **Median** | **IQR** |  |  |
| **IL-10 (median, IQR)** |  |  |  |  |  |  |  |  |
| Pre-op | 0.1 | (0.1,3.9) | 0.1 | (0.1,4.1) | 0.1 | (0.1,3.9) | Reference | - |
| 6-12 hours | 0.1 | (0.1,5.7) | 1.8 | (0.1,10.2) | 0.1 | (0.1,8.1) | 5.64 (2.35,8.93) | 0.001 |
| 48 hours | 2.9 | (2.2,4.8) | 2.7 | (1.3,3.6) | 2.9 | (1.7,4.1) | 3.45 (0.06,6.84) | 0.046 |
| Overall treatment effect |  |  |  |  |  |  | 3.37 (0.61,6.13) | 0.017 |
| **IL6 (median, IQR)** |  |  |  |  |  |  |  |  |
| Pre-op | 0.9 | (0.1,2.9) | 0.8 | (0.1,3.7) | 0.9 | (0.1,2.9) | Reference | - |
| 6-12 hours | 72.8 | (47.2,113.0) | 67.9 | (32.7,134.1) | 72.5 | (38.8,122.4) | 117.84 (79.63,156.05) | 0.000 |
| 48 hours | 32.1 | (1.6,72.8) | 50.2 | (25.2,80.5) | 37.8 | (3.9,79.3) | 70.82 (31.39,110.25) | 0.000 |
| Overall treatment effect |  |  |  |  |  |  | 8.96 (-26.47,44.38) | 0.620 |
| **IL8 (median, IQR)** |  |  |  |  |  |  |  |  |
| Pre-op | 1.9 | (0.0,3.8) | 2.0 | (0.0,5.0) | 1.9 | (0.0,4.2) | Reference | - |
| 6-12 hours | 12.0 | (6.6,23.1) | 15.0 | (8.7,33.4) | 14.4 | (7.8,25.8) | 21.24 (13.39,29.09) | 0.000 |
| 48 hours | 13.0 | (6.6,33.6) | 9.2 | (5.7,17.0) | 10.0 | (5.9,24.5) | 19.81 (11.73,27.90) | 0.000 |
| Overall treatment effect |  |  |  |  |  |  | 3.15 (-3.44,9.74) | 0.349 |
| **Troponin I (median, IQR)** |  | |  | |  |  |  |  |
| Pre-op | 26 | (11.5,51.5) | 34 | (14,60) | 32 | (13,55) | - | - |
| 6-12 hours post-op | 56 | (26,82) | 51.5 | (27,74) | 52.5 | (26.5,78.5) | - | - |
| 48 hours post-op | 1166.2 | (764.6,3149) | 2096 | (1201,3136.8) | 1634 | (889,3142.9) | - | - |
| Overall treatment effect |  |  |  |  |  |  | 21503.60  (-3557.25,46564.46) | 0.091 |
| **Urine NGAL (median, IQR)** |  |  |  |  |  |  |  |  |
| Pre-op | 5.1 | (2.6,14.3) | 5.8 | (2.0,17.2) | 5.7 | (2.2,15.6) | 0.14 (-0.04 , 0.32) | 0.138 |
| 24 hours | 45.8 | (28.5,59.2) | 50.3 | (27.2,59.1) | 46.0 | (27.4,59.2) | - | - |
| Overall treatment effect |  |  |  |  |  |  | -0.91 (-7.26 , 5.44) | 0.777 |
| **TIMP2*IGFBP (median, IQR)** |  |  |  |  |  |  |  |  |
| Pre-op | 0.2 | (0.1,0.2) | 0.1 | (0.1,0.2) | 0.1 | (0.1,0.2) | 0.27 (0.07 , 0.47) | 0.008 |
| 24 hours post-op | 0.2 | (0.1,0.3) | 0.2 | (0.1,0.2) | 0.2 | (0.1,0.3) | - | - |
| Overall treatment effect |  |  |  |  |  |  | -0.00 (-0.05 , 0.05) | 0.922 |

*Number of individuals contributing to each model by group and overall:*

**IL-10:** Overall: 123, Sildenafil: 57, Placebo: 66.

**IL-6:** Overall: 123, Sildenafil: 57, Placebo: 66.

**IL-8:** Overall: 123, Sildenafil: 57, Placebo: 66.

**Troponin I:** Overall: 77, Sildenafil: 34, Placebo: 43.

**Urine NGAL:** Overall: 118, Sildenafil: 55, Placebo: 63.

**TIMP2*IGFBP:** Overall: 117, Sildenafil: 55, Placebo: 62.

**Table S8: Non-fatal adverse events**

|  | **Randomised to Placebo (N=67)** | | | | **Randomised to Sildenafil**  **(N=58)** | | | |
| --- | --- | --- | --- | --- | --- | --- | --- | --- |
|  | **Adverse events** | | **SAEs** | | **Adverse events** | | **SAEs** | |
|  | **Events/patients** | **%** | **Events/patients** | **%** | **Events/patients** | **%** | **Events/patients** | **%** |
| **EXPECTED EVENTS (I.E. LISTED IN THE STUDY PROTOCOL)** | | | |  |  |  |  |  |
| STEMI* | 1 | 1.49 |  |  | 6 | 10.34 |  |  |
| Cardiac arrest* | 2 | 2.99 |  |  | 0 | 0.00 |  |  |
| SVT/AF** | 19 | 28.36 |  |  | 9 | 15.52 |  |  |
| VF/VT* | 1 | 1.49 |  |  | 1 | 1.72 |  |  |
| New pacing* | 1 | 1.49 |  |  | 3 | 5.17 |  |  |
| Use of inotropes* | 2 | 2.99 |  |  | 0 | 0.00 |  |  |
| Use of intra-aortic balloon pump* | 0 | 0.00 |  |  | 2 | 3.45 |  |  |
| Use of pulmonary artery catheter▪ | 18 | 26.87 |  |  | 15 | 25.86 |  |  |
| Use of vasoconstrictor | 52 | 77.61 |  |  | 45 | 77.59 |  |  |
| Low cardiac output* | 0 | 0.00 |  |  | 1 | 1.72 |  |  |
| Tracheostomy* | 0 | 0.00 |  |  | 0 | 0.00 |  |  |
| Mask CPAP* | 11 | 16.42 |  |  | 9 | 15.52 |  |  |
| Pneumothorax or effusion requiring draining▪▪ | 7 | 10.45 |  |  | 6 | 10.34 |  |  |
| Acute kidney injury* | 33 | 49.25 |  |  | 25 | 43.10 |  |  |
| AKIN stage 1 | 25 | 37.31 |  |  | 21 | 36.21 |  |  |
| AKIN stage 2 | 4 | 5.97 |  |  | 3 | 5.17 |  |  |
| AKIN stage 3 | 4 | 5.97 |  |  | 1 | 1.72 |  |  |
| Haemofiltration/dialysis since heart operation* | 4 | 5.97 |  |  | 1 | 1.72 |  |  |
| Peptic ulcer/ GI bleed/ perforation◦ | 0 | 0.00 |  |  | 1 | 1.72 |  |  |
| Other GI* | 3 | 4.48 |  |  | 3 | 5.17 |  |  |
| Permanent stroke* | 1 | 1.49 |  |  | 0 | 0.00 |  |  |
| TIA◦◦ | 0 | 0.00 |  |  | 1 | 1.72 |  |  |
| Excessive bleeding not requiring re-operation* | 6 | 8.96 |  |  | 6 | 10.34 |  |  |
| Wound dehiscence* | 1 | 1.49 |  |  | 2 | 3.45 |  |  |
| Reoperation¥ | 2 | 2.99 |  |  | 4 | 6.90 |  |  |

# Table S9: Detailed list of Serious Adverse Events by Treatment Group and Participant ID

- ***Placebo Group***

| Participant ID | Serious Adverse Event | Start date | End date | Frequency | Outcome | Treatment | Severity | Action taken | Related |
| --- | --- | --- | --- | --- | --- | --- | --- | --- | --- |
| 20 | Pneumonia | 29/04/2016 | 30/04/2016 | Single | Fatal | Concomitant Medication & Non-drug therapies | Fatal | None | Not related |
| 63 | Infective pericardial effusion | 11/05/2017 | 02/06/2017 | Continuous | Fatal | Concomitant Medication & Non-drug therapies | Fatal | None | Not related |
| 71 | Intermittent breathlessness | 02/07/2017 | 02/07/2017 | Single | Resolved | Concomitant Medication | Mild | None | Not related |

- ***Sildenafil Group***

| Participant ID | Serious Adverse Event | Start date | End date | Frequency | Outcome | Treatment | Severity | Action taken | Related |
| --- | --- | --- | --- | --- | --- | --- | --- | --- | --- |
| 115 | ECMO | 18/04/2018 | 20/04/2018 | Continuous | Fatal | Concomitant Medication & Non-drug therapies | Fatal | Study Discontinued | Not related |

**Supplementary Figure Legends**

**
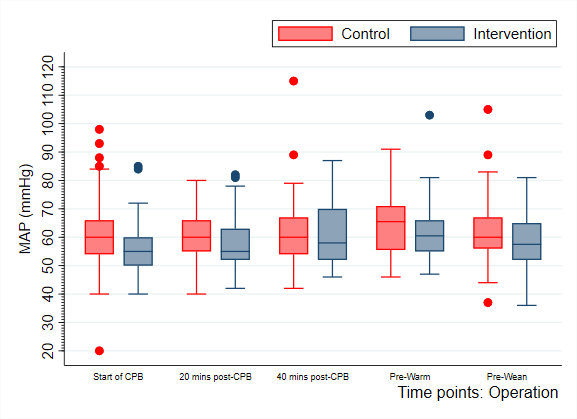
Figure S1. Panels show serial measurements of A. Blood Pressure, B. Haematocrit, C. Lactate**

A. Mean Arterial Blood Pressure


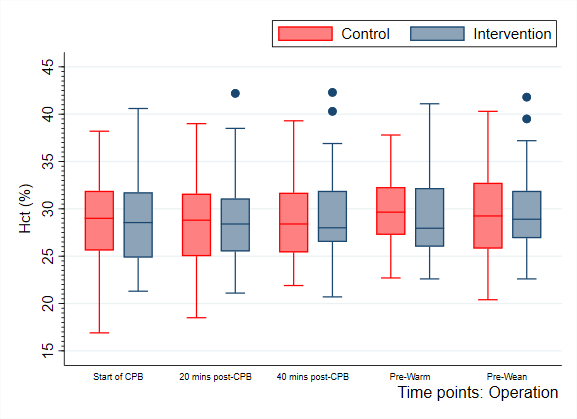
B. Haematocrit

**C.** **Lactate**

**
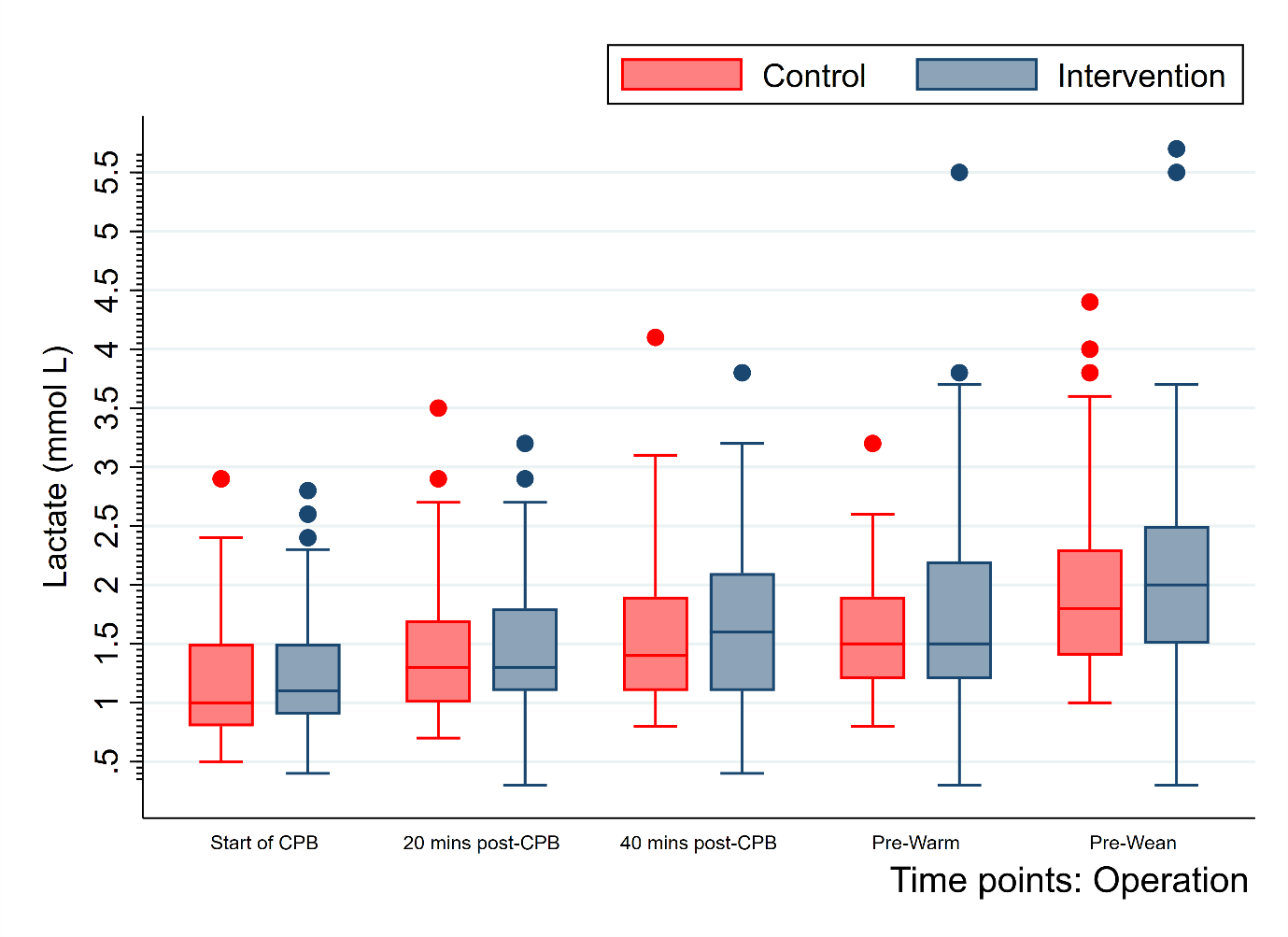
**

**Figure S2. MODS Scores Components**

1. Component: Respiratory

**
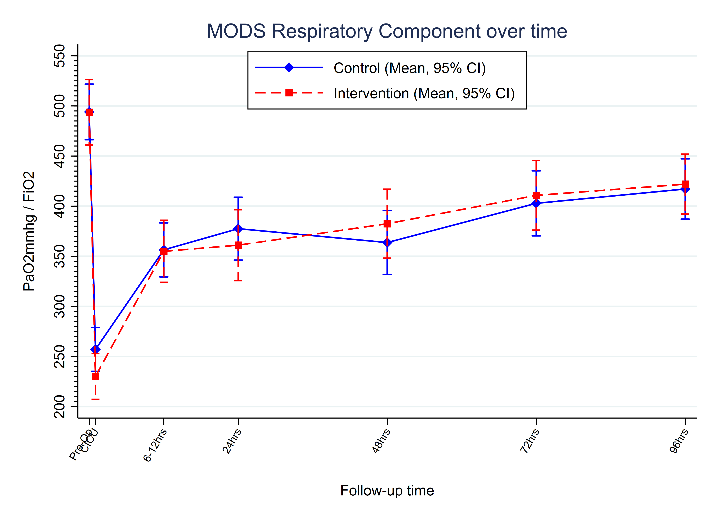
**

**
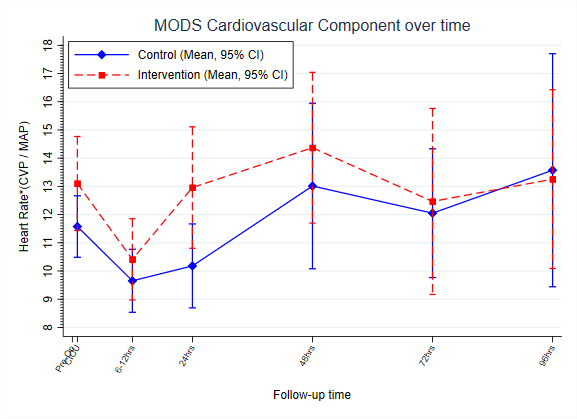
2. Component: Cardiovascular**

**
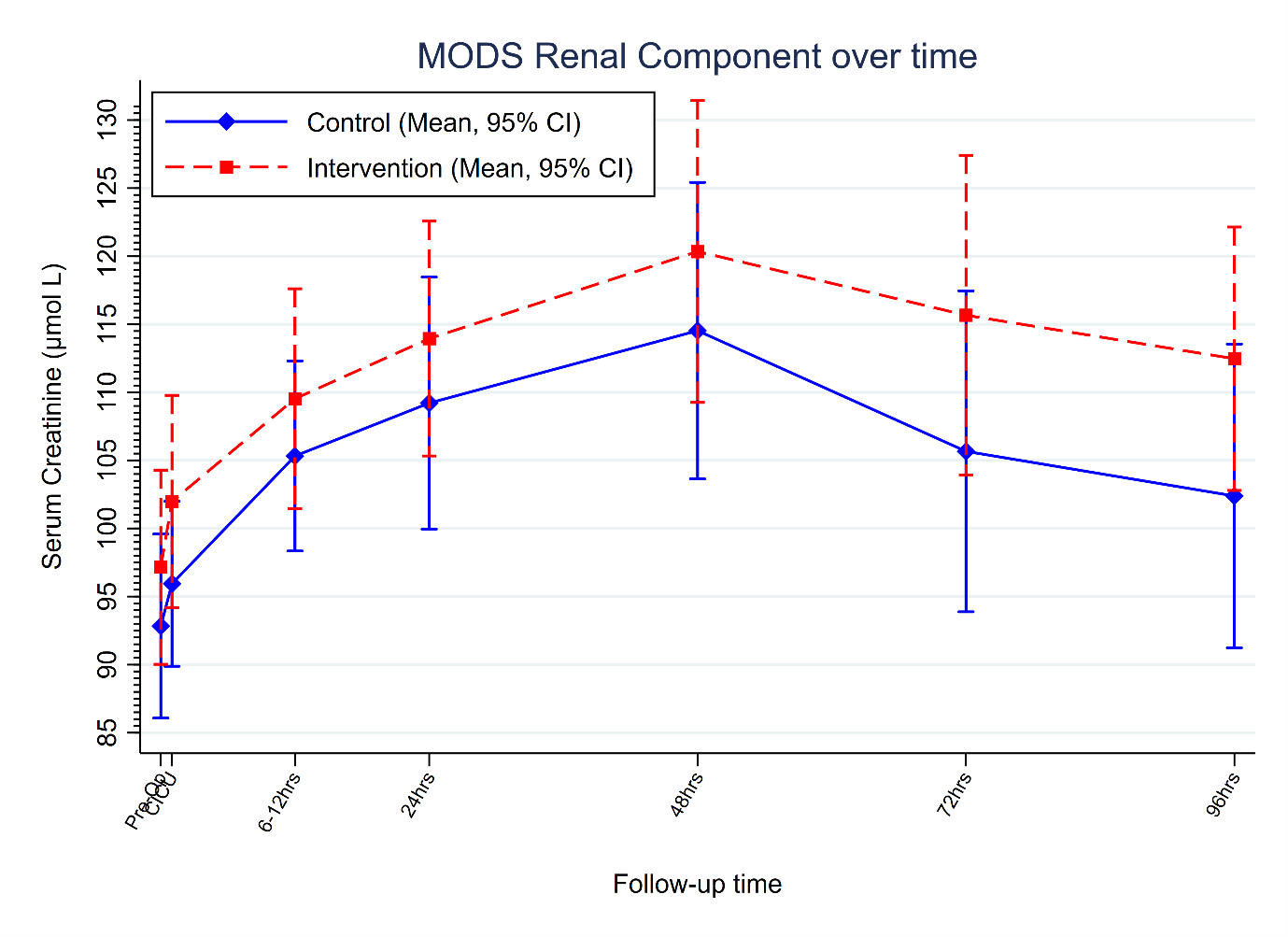
3. Component: Renal**

**4. Component: Hepatic**

**
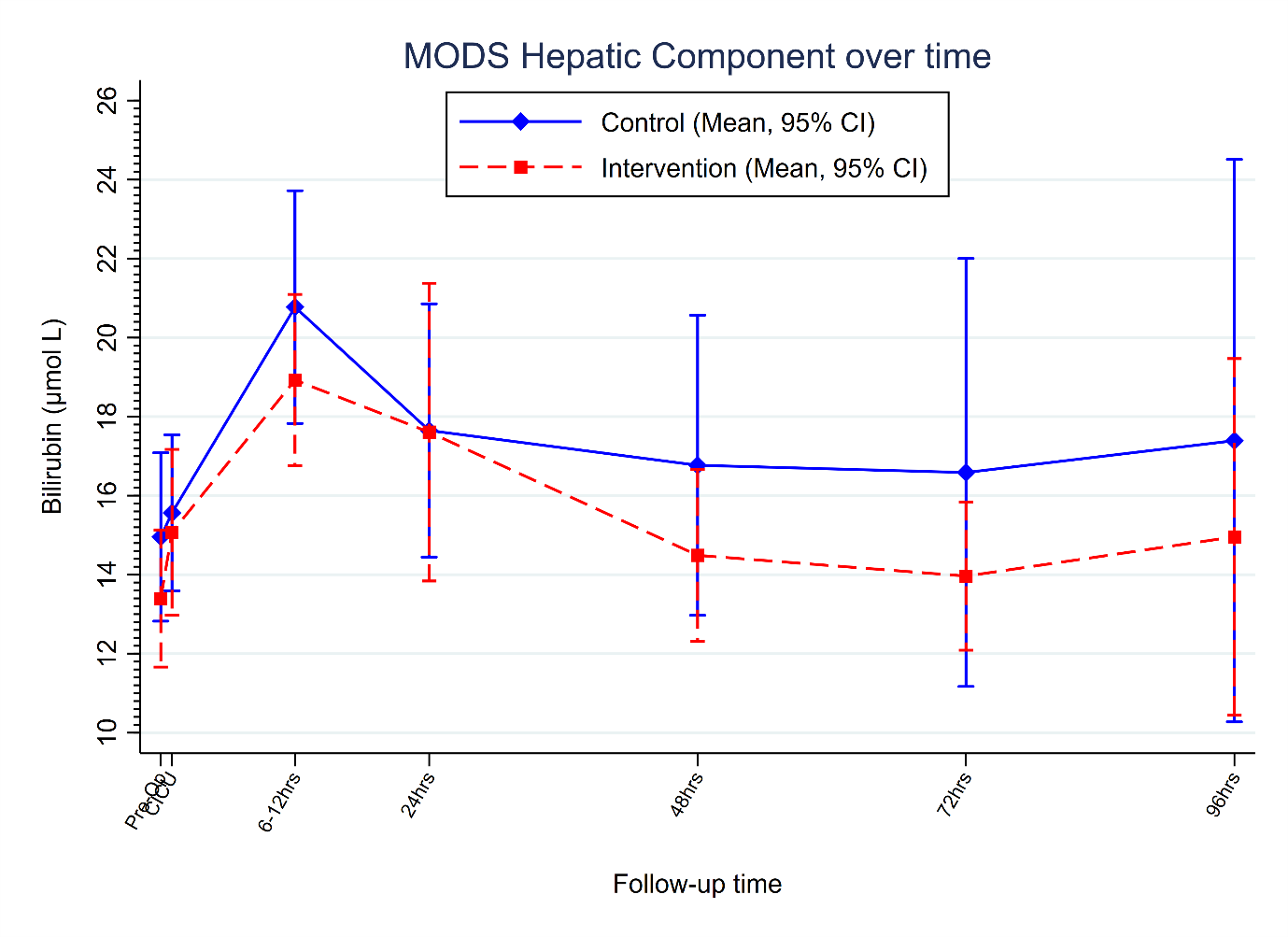
**

**5. Component: Hematologic**

**
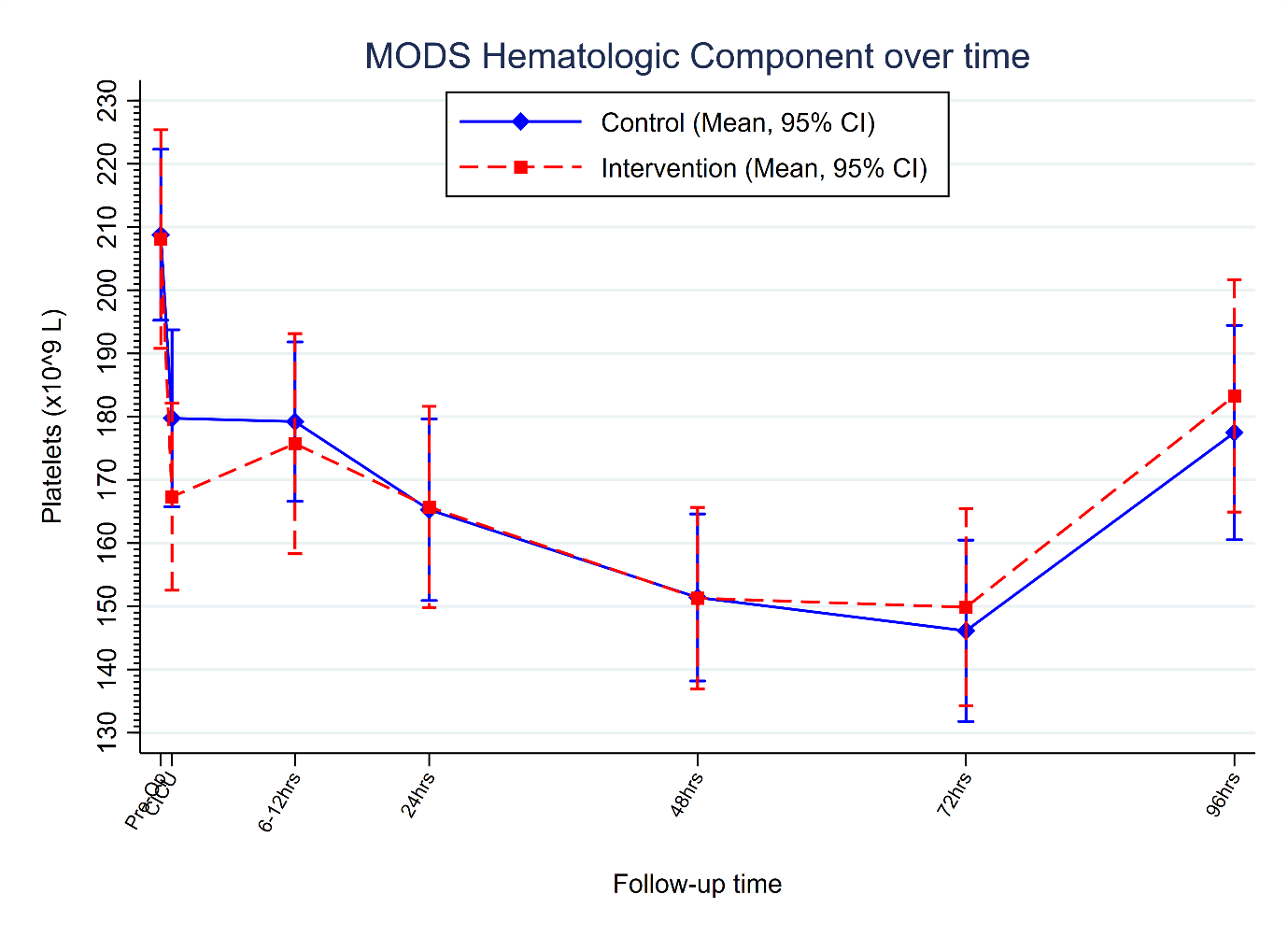
**

**6. Component: Neurologic**

**NB:** *the Neurologic component has not been described as all 125 patients have missing data across all time points for the Glasgow Coma Scale (GCS) measurement.*

**Figure S3. Secondary Outcomes**: **A.** Time to discharge from ICU. **B.** Time to discharge from Hospital Unit.


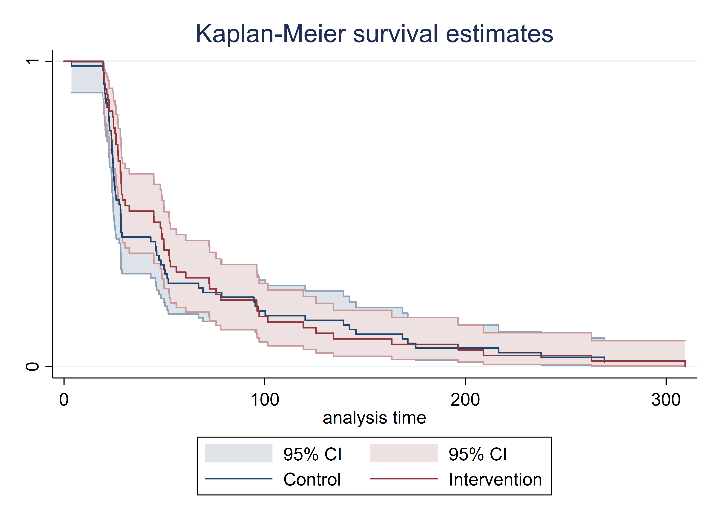
A. Time to discharge from ICU


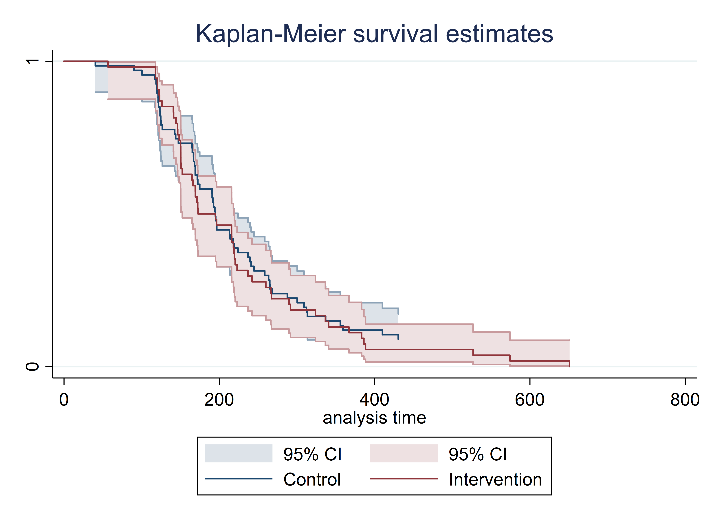
**B. Time to discharge from Hospital Unit**

**Details of Contributors**

All of the study authors, external and internal, had full access to all of the data (including statistical reports and tables) in the study and can take responsibility for the integrity of the data and the accuracy of the data analysis. Individual contributions to the study were as follows:

**List of Investigators and research team members are as follows:**

**Data Monitoring and Safety Committee**

Prof. Derek Hausenloy, Cardiovascular and Metabolic Disorders Program, Duke-NUS Graduate Medical School, Singapore (Chairman)

Dr. Nick Selby, Consultant Nephrologist, Royal Derby Hospital (Member – Independent Clinician)

Prof. Chris Imray, Consultant Vascular and Transplant Surgeon, University Hospitals Coventry and Warwickshire NHS Trust (Member – Independent Clinician)

Mr. Richard Akers (Member – Lay)

Prof. Chris Jennison, Professor of Statistics, University of Bath (Member – Independent Statistician)

**Patient and Public Involvement Group members**

Phil Caldwell

Ian Shuttlewood

Anthony Locke

Paul Haywood

Jagruti Lalseta

Alan Phillips

Terry Finnigan

Peter Read

David Mayer

# References

1. KDIGO A, Work, Group,: KDIGO clinical practice guideline for acute kidney injury. Kidney International Supplement 2012; 2: 1-138

2. Levey AS, Berg RL, Gassman JJ, Hall PM, Walker WG: Creatinine filtration, secretion and excretion during progressive renal disease. Modification of Diet in Renal Disease (MDRD) Study Group. Kidney Int Suppl 1989; 27: S73-80

3. Ferguson ND, Fan E, Camporota L et al: The Berlin definition of ARDS: an expanded rationale, justification, and supplementary material. Intensive Care Med 2012; 38: 1573-82

4. Marshall JC, Cook DJ, Christou NV, Bernard GR, Sprung CL, Sibbald WJ: Multiple organ dysfunction score: a reliable descriptor of a complex clinical outcome. Crit Care Med 1995; 23: 1638-52

5. Nohria A, Gerhard-Herman M, Creager MA, Hurley S, Mitra D, Ganz P: Role of nitric oxide in the regulation of digital pulse volume amplitude in humans. J Appl Physiol (1985) 2006; 101: 545-8

**CONSORT 2010 checklist of information to include when reporting a randomised trial***

| **Section/Topic** | **Item No** | **Checklist item** | **Reported on page No** |
| --- | --- | --- | --- |
| **Title and abstract** | | | |
|  | 1a | Identification as a randomised trial in the title | 1 (e-supplement) |
|  | 1b | Structured summary of trial design, methods, results, and conclusions (for specific guidance see CONSORT for abstracts) | 2 (e-supplement) |
| **Introduction** | | | |
| Background and objectives | 2a | Scientific background and explanation of rationale | 3 (e-supplement) |
|  | 2b | Specific objectives or hypotheses | 3 (e-supplement) |
| **Methods** | | | |
| Trial design | 3a | Description of trial design (such as parallel, factorial) including allocation ratio | 4 (e-supplement) |
|  | 3b | Important changes to methods after trial commencement (such as eligibility criteria), with reasons | 7 (e-supplement) |
| Participants | 4a | Eligibility criteria for participants | 4 (e-supplement) |
|  | 4b | Settings and locations where the data were collected | 5 (e-supplement) |
| Interventions | 5 | The interventions for each group with sufficient details to allow replication, including how and when they were actually administered | 4 (e-supplement) |
| Outcomes | 6a | Completely defined pre-specified primary and secondary outcome measures, including how and when they were assessed | 5 (e-supplement) |
|  | 6b | Any changes to trial outcomes after the trial commenced, with reasons | 7 (e-supplement) |
| Sample size | 7a | How sample size was determined | 6 (e-supplement) |
|  | 7b | When applicable, explanation of any interim analyses and stopping guidelines | N/A |
| Randomisation: |  |  |  |
| Sequence generation | 8a | Method used to generate the random allocation sequence | 3 (e-supplement) |
|  | 8b | Type of randomisation; details of any restriction (such as blocking and block size) | 3 (e-supplement) |
| Allocation concealment mechanism | 9 | Mechanism used to implement the random allocation sequence (such as sequentially numbered containers), describing any steps taken to conceal the sequence until interventions were assigned | 3 (e-supplement) |
| Implementation | 10 | Who generated the random allocation sequence, who enrolled participants, and who assigned participants to interventions | 3 (e-supplement) |
| Blinding | 11a | If done, who was blinded after assignment to interventions (for example, participants, care providers, those assessing outcomes) and how | 3 (e-supplement) |
|  | 11b | If relevant, description of the similarity of interventions | N/A |
| Statistical methods | 12a | Statistical methods used to compare groups for primary and secondary outcomes | 5-6 (main manuscript) |
|  | 12b | Methods for additional analyses, such as subgroup analyses and adjusted analyses | 5-6 (main manuscript) |
| **Results** | | | |
| Participant flow (a diagram is strongly recommended) | 13a | For each group, the numbers of participants who were randomly assigned, received intended treatment, and were analysed for the primary outcome | diagram attached |
|  | 13b | For each group, losses and exclusions after randomisation, together with reasons | diagram attached |
| Recruitment | 14a | Dates defining the periods of recruitment and follow-up | 7 (main manuscript) |
|  | 14b | Why the trial ended or was stopped | N/A |
| Baseline data | 15 | A table showing baseline demographic and clinical characteristics for each group | 14-17 (main manuscript) |
| Numbers analysed | 16 | For each group, number of participants (denominator) included in each analysis and whether the analysis was by original assigned groups | 18 (main manuscript) |
| Outcomes and estimation | 17a | For each primary and secondary outcome, results for each group, and the estimated effect size and its precision (such as 95% confidence interval) | 18 (main manuscript) and 16-19 (e-supplement) |
|  | 17b | For binary outcomes, presentation of both absolute and relative effect sizes is recommended | N/A |
| Ancillary analyses | 18 | Results of any other analyses performed, including subgroup analyses and adjusted analyses, distinguishing pre-specified from exploratory | 14-16 (e-supplement) |
| Harms | 19 | All important harms or unintended effects in each group (for specific guidance see CONSORT for harms) | 22-24 (e-supplement) |
| **Discussion** | | | |
| Limitations | 20 | Trial limitations, addressing sources of potential bias, imprecision, and, if relevant, multiplicity of analyses | 9 (main manuscript) |
| Generalisability | 21 | Generalisability (external validity, applicability) of the trial findings | 10 (main manuscript) |
| Interpretation | 22 | Interpretation consistent with results, balancing benefits and harms, and considering other relevant evidence | 10 (main manuscript) |
| **Other information** | | |  |
| Registration | 23 | Registration number and name of trial registry | 1, 2, 4 (main manuscript) |
| Protocol | 24 | Where the full trial protocol can be accessed, if available | 4 (main manuscript) |
| Funding | 25 | Sources of funding and other support (such as supply of drugs), role of funders | 11 (main manuscript) |

*We strongly recommend reading this statement in conjunction with the CONSORT 2010 Explanation and Elaboration for important clarifications on all the items. If relevant, we also recommend reading CONSORT extensions for cluster randomised trials, non-inferiority and equivalence trials, non-pharmacological treatments, herbal interventions, and pragmatic trials. Additional extensions are forthcoming: for those and for up to date references relevant to this checklist, see [www.consort-statement.org](http://www.consort-statement.org).
